# A bioinformatic analysis identifies circadian expression of splicing factors and time-dependent alternative splicing events in the HD-MY-Z cell line

**DOI:** 10.1038/s41598-019-47343-w

**Published:** 2019-07-30

**Authors:** Nikolai Genov, Alireza Basti, Mónica Abreu, Rosario Astaburuaga, Angela Relógio

**Affiliations:** 1 Charité - Universitätsmedizin Berlin, corporate member of Freie Universität Berlin, Humboldt - Universität zu Berlin, and Berlin Institute of Health, Institute for Theoretical Biology, Berlin, Germany; 2 Charité - Universitätsmedizin Berlin, corporate member of Freie Universität Berlin, Humboldt - Universität zu Berlin, and Berlin Institute of Health, Medical Department of Hematology, Oncology, and Tumor Immunology, Molecular Cancer Research Center, Berlin, Germany

**Keywords:** Gene regulatory networks, Microarrays

## Abstract

The circadian clock regulates key cellular processes and its dysregulation is associated to several pathologies including cancer. Although the transcriptional regulation of gene expression by the clock machinery is well described, the role of the clock in the regulation of post-transcriptional processes, including splicing, remains poorly understood. In the present work, we investigated the putative interplay between the circadian clock and splicing in a cancer context. For this, we applied a computational pipeline to identify oscillating genes and alternatively spliced transcripts in time-course high-throughput data sets from normal cells and tissues, and cancer cell lines. We investigated the temporal phenotype of clock-controlled genes and splicing factors, and evaluated their impact in alternative splice patterns in the Hodgkin Lymphoma cell line HD-MY-Z. Our data points to a connection between clock-controlled genes and splicing factors, which correlates with temporal alternative splicing in several genes in the HD-MY-Z cell line. These include the genes *DPYD*, *SS18*, *VIPR1* and *IRF4*, involved in metabolism, cell cycle, apoptosis and proliferation. Our results highlight a role for the clock as a temporal regulator of alternative splicing, which may impact malignancy in this cellular model.

## Introduction

Pre-mRNA splicing is a fundamental biological process through which the introns of a nascent RNA are removed and exons are merged to form a mature RNA that is then translated into a protein. Splicing is catalysed by a large ribonucleoprotein (RNP) complex – the spliceosome^1^. This macromolecular complex is guided by *cis*-acting sequences on the pre-mRNA (splice sites, exonic and intronic splicing enhancers and silencers) and interacts with *trans*-acting elements (splicing factors - SF) to excise the pre-mRNA at the intron/exon junctions (so-called splice junctions). Notably, up to 94% of human genes undergo differential splicing and thus exhibit various alternative-spliced isoforms giving rise to a variety of proteins from the same pre-mRNA^2^. Hence, via alternative splicing (AS) different proteins are generated, resulting in a greater variety of cellular functions and pathways. AS has been reported to be involved in the specification of several features of protein function including their cellular localization, enzymatic properties and their interaction with ligands, as well as their role in gene expression regulation and histone modifications^3^.

Like many cellular processes, AS is differentially regulated during cellular growth and tissue development^4^. This plasticity is also of advantage during tumorigenesis as it enables cancer cells to produce proteins that suit their proliferative and metastatic needs. By using AS, cancer cells benefit from isoforms specific to particular stages of oncogenesis and disease development^5–7^. The dysregulation of AS appears to affect almost all aspects of tumour biology, including cell cycle control, metastasis, metabolism, invasion and angiogenesis^5,8^. Furthermore, published work has revealed cancer-specific altered splicing patterns in various cancer types (e.g. Hodgkin lymphoma, colon cancer and lung cancer) and identified several splicing factors that act as oncoproteins and contribute to tumour initiation (e.g. hnRNP E1, SF2/ASF and SRSF6)^9–16^. In particular, the analysis of AS using microarray data revealed stage–specific AS patterns between cell lines derived from Hodgkin lymphoma (HL) tumours at stage IIIb (HD-MY-Z) and IV (HDML-2) that are distinguishable from a non-tumour B lymphoblastoid cell line (LCL-HO) and from HL tumours of different stages^11^. Additionally, several splicing factors (e.g. hnRNP E1, U2AF65 and NOVA-1/2) are differentially expressed between HL cell lines and the B lymphoblastoid cell line LCL-HO. Accordingly, HL tumours from the same stage (HDML-2 and L-540, stage IV) show greater similarity in their splicing pattern (70%) when compared to HL stage III cell line (~55%)^11^.

Recent studies in mice showed a correlation between certain SF and the functionality of the clock machinery^17,18^. Furthermore, specific RNA-binding proteins involved in AS (e.g. LARK, hnRNP Q, CIRP, 4E-Bp1) were reported to regulate the expression of core-clock components in terms of mRNA localization, stability, and translation^19–22^. Conversely, several co-transcriptional and post-transcriptional processes have been reported to be regulated in a circadian manner in mammals, including AS, polyadenylation, mRNA stability and transport^23,24^. These findings point to a bidirectional interplay between the components and mechanisms of splicing and the circadian clock. Numerous studies have illustrated the role of the mammalian circadian clock in key cellular pathways and functions (such as the cell cycle^25^, metabolism^26^ and the immune system^27^) and revealed a prominent role of clock disruption in tumorigenesis^26,28,29^. Despite the increasing relevance of AS in cancer onset and progression, the role of the circadian clock as an intermediary component in this process remains unknown, and only a few studies have investigated the circadian expression phenotype of splicing factors and their impact in AS^18,23,30^.

In this study, we analysed time-course microarray data sets to identify oscillating genes, and to investigate the expression of SF in different tissue types and cell lines from mouse and human cell lines. In addition, we used a recently published^31^ time-course data set for the HD-MY-Z cell line, for which stage-specific patterns of AS have previously been described^11^, as a cellular model system to investigate a possible correlation between the circadian clock and the splicing machinery. We found connections between the core-clock and clock-controlled genes and a pre-defined set of SF, by means of protein-protein interaction networks. Additionally, we analysed the extent of AS events in the HD-MY-Z cell line in a time-dependant manner and identified several genes, including *DPYD* (dihydropyrimidine dehydrogenase), *IRF4* (interferon regulatory factor 4), *POP4* (POP4 homolog, ribonuclease P/MRP subunit), *SS18* (nBAF chromatin remodelling complex subunit), *MAGOH* (mago homolog, exon junction complex subunit) *and VIPR1* (vasoactive intestinal peptide receptor 1), involved in the cell cycle, apoptosis, drug metabolism and RNA processing and splicing that show expression patterns resulting from a temporal AS. Our results emphasize the existence of a putative circadian regulation of SF that subsequently may impact on AS decisions potentially relevant in cancer onset and or progression.

## Results

### A network analysis reveals distinct interacting clusters among splicing factors involved in various cellular pathways

**Figure 1:**
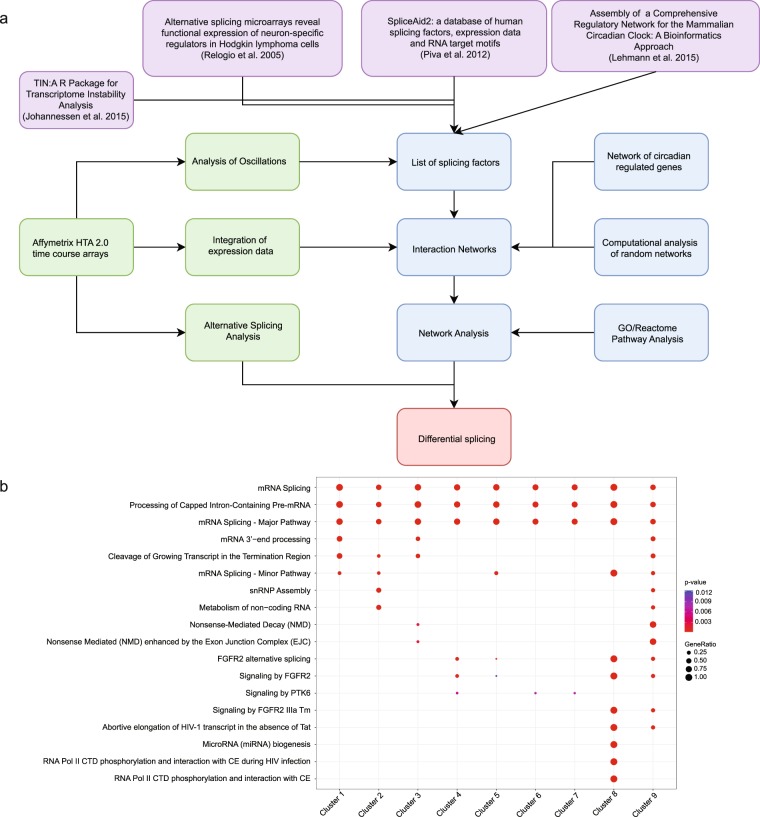
The splicing factors form a tightly interconnected network with additional non-splicing functionalities. (**a**) Pipeline of the analysis approach for the detection of differential splicing. The original sources of data such as databases and publications are coloured in purple. The steps required for the creation of protein-protein interaction networks are coloured in blue. The array analysis steps are coloured in green. The resulting differential splicing is coloured in red. (**b**) The resulting nine clusters were investigated for the enrichment of Reactome pathways (*p*-values are indicated).

To analyse the putative regulation of RNA splicing processes via the circadian clock we started by compiling a comprehensive list of splicing factors (SF). The SF-list merges data from different sources: the SpliceAid2^32^ database, the TIN R package^33^ and from a previous publication by Relógio *et al*.^11^. This resulted in a list of 341 splicing factors (Supp. Table S1), which we used for further analysis as input to an integrated pipeline as indicated in the Fig. 1a.

The pipeline involves the analysis of time-course high-throughput data obtained with the splicing-sensitive Affymetrix HTA-2.0 arrays, a bioinformatic analysis by protein-protein interaction networks, the Reactome pathway enrichment of genes for which changes in alternative splicing behaviour are detected. We also include an analysis for enrichment of transcription factors binding sites in the promoter regions of the splicing factors, and the computational evaluation of alternative splicing events, as well as their visualization. First, we used the SF list to explore the correlations among its elements, by creating a network of SF based on protein-protein interaction data from the IntAct database. The data retrieved from the database forms one large connected component containing a total of 237 nodes and 898 edges. A clustering based on the interactions between the nodes using a community-clustering algorithm (GLay) resulted in nine clusters (Supp. Fig. S1 and Supp. Table S2). We further analysed the functional relevance of the clusters by performing a comparative enrichment analysis for Reactome pathways (Supp. Table S3), which yielded 18 pathway clusters (*p* < 0.05) (Fig. 1b). Splicing related terms are found in all clusters (e.g. mRNA splicing, Processing of Capped Intron-Containing Pre-mRNA), which justifies our selection of SFs and strengthens their specificity. The enrichment also highlights additional functional diversity among the clusters of SF. Cluster 8 shows a strong correlation to RNA Polymerase II (*p* = 1.018E-19) and nonsense-mediated decay (NMD) appears enriched for two of the clusters (Cluster 9 and 3, *p* = 1.626E-11 and 2.371E-02 respectively), a process which is involved in the degradation of aberrant mRNA isoforms. Additionally, FGFR2 (fibroblast growth factor receptor 2) signalling or AS of FGFR2 is enriched in clusters 4, 8 and 9. FGFR2 pathway is known for its role in cancer. It cross-talks (via activation or inhibition) with various signalling pathways, including WNT, Notch and Hedgehog involved in tissue development and cellular proliferation^34^. Altogether, our enrichment analysis points to the involvement of the selected SF in diverse cellular functions beyond splicing or splicing related terms.

### Oscillations in elements of the clock network correlate with oscillations in splicing factors in healthy and cancer samples

To investigate in more detail the existence of a putative interplay between the circadian clock and the SF, we performed an analysis of the interactions between elements of the predefined network of SF and elements of the network of circadian regulated genes (NCRG) previously published by our group^35^. For this network analysis, we used the data from the IntAct database (snapshot from 2015 present in iRefIndex^36^). We identified 162 direct interactions based on the IRefIndex data (snapshot from 2015) between the SF and the NCRG sets, while a comparison of 100 randomly generated networks produced 34.7 ± 25.7 (mean ± SD) interactions between the SF set and gene sets of the same size as the NCRG. Finally, we produced a detailed network using the data retrieved from the current version of IntAct reduced to interacting nodes for the SF and the NCRG sets with Cytoscape (Supp. Fig. S2). The network contains 130 NCRG and 251 SF elements, in a total of 186 connections (edges) between both sets. Additionally, 177 edges exist within the NCRG and 873 edges within the SF elements. In average each SF is connected to 0.74 NCRG elements and each NCRG element has in average 1.43 connected SF. The elements with the highest degree of connections are CSNK2A1, MATR3 and WDR5 for the NCRG and SRPK2, EIF4A3 and RNPS1 for the SF sets. CSNK2A1 (Casein kinase II subunit alpha) is involved in numerous cellular processes, such as cell cycle control and apoptosis^37^. Additionally, CSNK2A1 plays an important role in the circadian clock function by phosphorylating ARNTL (BMAL1) and thus controlling CLOCK nuclear entry^38^. MATR3 (Matrin-3) is a RNA binding protein involved in micro RNA regulation^39^ and activation of innate immune response^40^. WDR5 (WD repeat-containing protein 5) contributes to histone modification and (mainly of histone H3) and is therefore involved in epigenetic transcriptional activation^41^. From the set of SF elements, SRPK2 (SRSF protein kinase 2) is involved in the phosphorylation of SR splicing factors and in the process of spliceosome assembly and thus in the regulation of splicing^42^. EIF4A3 (Eukaryotic initiation factor 4A-III) is an ATP-dependant RNA helicase, which belongs to the spliceosome complex and is involved in pre-mRNA splicing^43,44^. RNPS1 (RNA-binding protein with serine-rich domain 1) is part of pre-splicing multiprotein RNP complex involved in RNA splicing^45^. Additionally, RNPS1 mediates NMD via interacting with the UPF complex^46^.

**Figure 2:**
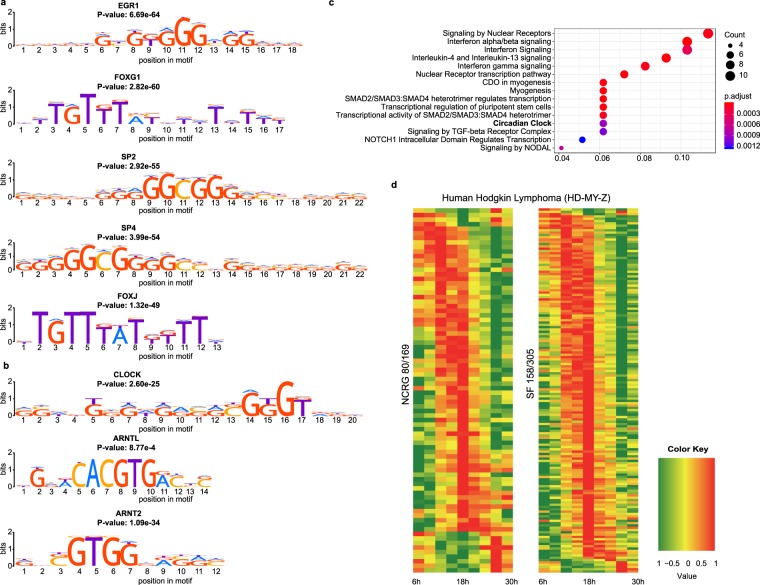
A bi-directional interplay between the splicing factors and the NCRG is achieved through direct protein-protein interactions and transcriptional regulation. (**a**) We detected 231 enriched motifs for binding sites with the MEME Suite AME tool for 184 unique transcription factors from the HOCOMOCOv10 database. The motifs and adjusted *p*-values for the top 5 scoring TFs are shown. (**b**) The promotor region of SF contains significantly (ARNTL *p* = 8.77E-04, ARNT2 *p* = 1.09E-34, CLOCK *p* = 2.60E-25) enriched transcription factor binding sites for several core-clock and clock-related genes. (**c**) Enrichment of Reactome pathways for the TFs with enriched binding sites in the promoter regions of the SF. The gene ratio denotes the ratio between the number of the genes associated with the term and the total number of genes associated with the term in the pathway. (**d**) Oscillations of the NCRG and splicing factors in Hodgkin Lymphoma (HD-MY-Z). The total number of genes for the NCRG and the SF is derived after mapping to human Entrez Gene IDs.

The high number of interactions between the NCRG and the SF, as compared to the average of the random sets, led us to investigate the existence of an enrichment of transcription factors (TF) binding sites, within the promoter regions of SF elements, as defined in the HOCOMOCO v10^47^ database. We searched for TF binding sites of clock genes in the promoter regions of the SF, defined as the 2kbp upstream region, which yielded 231 enriched transcription factor binding site motifs mapped to 184 unique genes (Supp. Table S4). The total top scoring TFs are depicted in Fig. 2a. Our analysis identified central clock genes (*CLOCK*, *p* = 2.60E-25; *ARNTL* (*BMAL1*), *p* = 8.77E-4), as well as clock-related genes (*ARNT2 - Aryl Hydrocarbon Receptor Nuclear Translocator 2,p* = 1.09E-34) (Fig. 2b) to significantly bind to the promoter regions of the SFs as part of the total 184 detected unique TFs. We identified a total of 8 NCRG elements with enriched binding sites in the promoter regions of the splicing factors (AHR, ATF2, CLOCK, SMAD4, TEF, IRF7, SP1, ARNTL). AHR (aryl hydrocarbon receptor) is a Ligand-activated transcriptional activator involved in the cell cycle^48^. It also regulates the circadian clock by inhibiting the circadian expression of *PER1* via repressing the CLOCK-ARNTL (BMAL1) heterodimer mediated transcriptional activation of *PER1*^49^. ATF2 (cyclic AMP-dependent transcription factor ATF-2) regulates the transcriptional activation of various genes including those involved in cell growth and DNA damage response (e.g. ATM, Chk1 and Chk2)^50^. TEF (thyrotroph embryonic factor) is a transcription factor as well, which binds to a minimal DNA-binding sequence. IRF7 (interferon regulatory factor 7) is a transcriptional regulator of type I interferon (IFN)-dependent immune responses, which plays a critical role in the innate immune response. It regulates the transcription of type I IFN genes (IFN-alpha and IFN-beta) and IFN-stimulated genes (ISG)^51^. SP1 (transcription factor Sp1) regulates the expression of a large number of genes involved in a variety of processes such as cell growth, apoptosis, differentiation and immune responses^52–55^. Additionally, SP1 was reported to positively regulate the transcription of the core-clock component ARNTL (BMAL1)^56^. This reinforces our initial hypothesis regarding an interplay between the circadian clock and the circadian regulation of the expression of splicing factors. The TF with lowest *p*-value detected for the promoter regions of the SF was *EGR1* (Early Growth Response 1) (adjusted BH *p* = 6.69E-64) involved in the regulation of differentiation. *EGR1* was shown to be activated by the CLOCK/BMAL1 heterodimer (via E-box elements) in mouse and regulates the transcription of several core-clock genes, including *Bmal1*, *Per1*, *Per2* and *Nr1d1* and *Nr1d2*^57^. Interestingly, a pathway enrichment analysis of the identified TFs with enriched binding site motifs reveals that the circadian clock pathway is highly enriched (adjusted BH *p* = 8.9767E-03), which underlines the connection of the TFs to the circadian clock (Fig. 2c, Supp. Table S5).

To further explore the regulation of splicing via the circadian clock, we investigated the expression phenotype of the circadian clock and SF elements in six high-throughput time-course datasets for human cell lines and mouse cells and tissues (all data sets are available from public repositories and their accession numbers provided in Materials and Methods).

The datasets consist of mouse macrophages (Supp. Fig. S3, GSE25585), mouse NIH3T3 fibroblasts (Supp. Fig. S3, GSE11922), mouse pituitary gland^58^ (Supp. Fig. S3) and mouse liver (Supp. Fig. S3, GSE11923), the cancer cell lines U2OS (human osteosarcoma, time course data set^58^, GSE13949, Fig. 2d) and HD-MY-Z (human Hodgkin Lymphoma cell line, time course data set^27^, ArrayExpress E-MTAB-6400, Supp. Fig. S4). The expression patterns of the circadian clock (represented by the NCRG) and SF elements in these high-throughput time-course datasets show a strong similarity between the two sets for all cell lines/tissues (Supp. Figs S3 and S4). This similarity is also apparent in the time-course high-throughput expression dataset for the human Hodgkin Lymphoma cell lines HD-MY-Z produced by our group (Fig. 2d). Additionally, we investigated the intersect of the SF oscillating with a period of 24 ± 3 h for the mouse and for the human samples, oscillations were detected using Metacycle^59^, a specialized R package for the investigation of oscillating behaviour from high-throughput data. For the mouse samples we detected an intersect of 9 splicing factors: *Hnrnpd*, *Khdrbs1*, *Srpk2*, *Wac*, *Tardbp*, *Sf3a1*, *Puf60*, *Rbm28*, *Prpf31* (Supp. Fig. S3e). Most of the detected splicing factors (*Hnrnpd*, *Khdrbs1*, *Tardbp* and *Puf60*) are DNA/RNA binding proteins involved in various steps of RNA biogenesis and processing, as well as several nuclear processes (such as pre-mRNA splicing)^60^. Sf3a1, Rbm28 and Prpf31 belong to the spliceosome ribonucleoprotein complexes and are thus involved in pre-mRNA splicing^61,62^. Wac acts as a linker between gene transcription and histone H2B monoubiquitination which positively regulates MTOR activity^63^.

For the HD-MY-Z and U2OS cell lines the intersect results in 24 genes (Supp. Fig. S4) including *NONO* (U2OS, *p* = 1.178-E05; HD-MY-Z, *p* = 3.585E-03) and *POLR2A* (U2OS, *p* = 2.902E-04; HD-MY-Z, *p* = 1.637E-02). NONO (non-POU domain containing octamer binding) is involved in the regulation of the circadian clock and cell cycle via interactions with PER and p16-INK4A (respectively) and acts as a coupling element between both processes^25,64^. POLR2A (RNA polymerase II subunit A) is a DNA-dependent RNA polymerase involved in the transcription of DNA to RNA^65^. Notably, mutations in *POLR2A* were reported to drive neoplasia in meningioma^66^ and play a potential role as a therapy target in various cancer types, including colorectal cancer^67^. Subsequently, we retrieved the sets of oscillating genes and carried out comparisons to determine a possible correlation between the number of oscillating genes in the NCRG set and the number of oscillating genes in the SF set, for all datasets. Our data shows a spearman correlation of 0.986 between the numbers of oscillating genes from the NCRG and the SF from all datasets analysed. In particular, the percentages of oscillating genes in the SF and the NCRG follow a similar pattern across tissues and cell lines, as shown in Supplementary Fig. S4, further underlining the connection between the NCRG and the SF.

**Figure 3:**
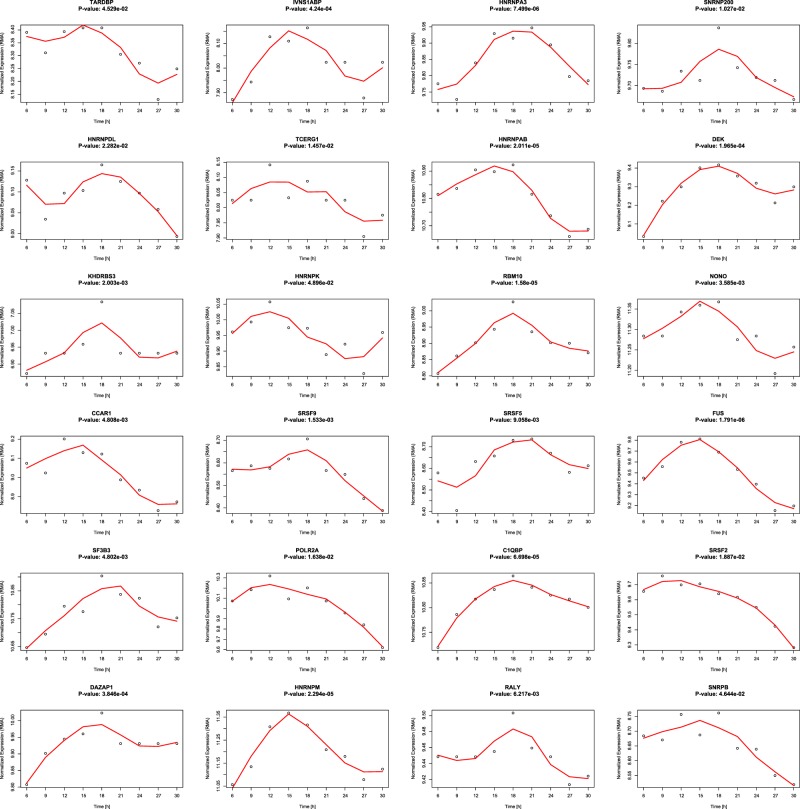
The expression of the 24 splicing factors detected to oscillate in human samples shows marked changes over time. We detected the oscillations of the genes through high-throughput analysis with MetaCycle. *p*-values given are for periods between 21 and 27 hours.

We further investigated in detail the 24 splicing factors for which oscillations are present both in the U2OS and the HD-MY-Z cell line. The expression of the 24 splicing factors derived from the HTA 2.0 arrays of our time course is shown in Fig. 3 together with the *p*-values calculated with MetaCycle and summarized from the three methods implemented in the package – ARS, JTK and LS. The expression pattern was also used to correlate the oscillating splicing factors to the genes for which alternative splicing and oscillation over time are detected (Supp. Fig. S5). We show the 9 splicing factors for which oscillations are present in all mouse tissues as Supplementary Fig. S3.

Additionally, we performed a correlation analysis for the 24 SF and elements of the clock network. This analysis points toward a widespread positive correlation between the expression of SF and the expression of the core-clock genes with the exception of a negative correlation between RORB/RORC and most other genes from the two sets.

### The circadian clock affects splicing and induces temporal changes in splicing events

**Figure 4:**
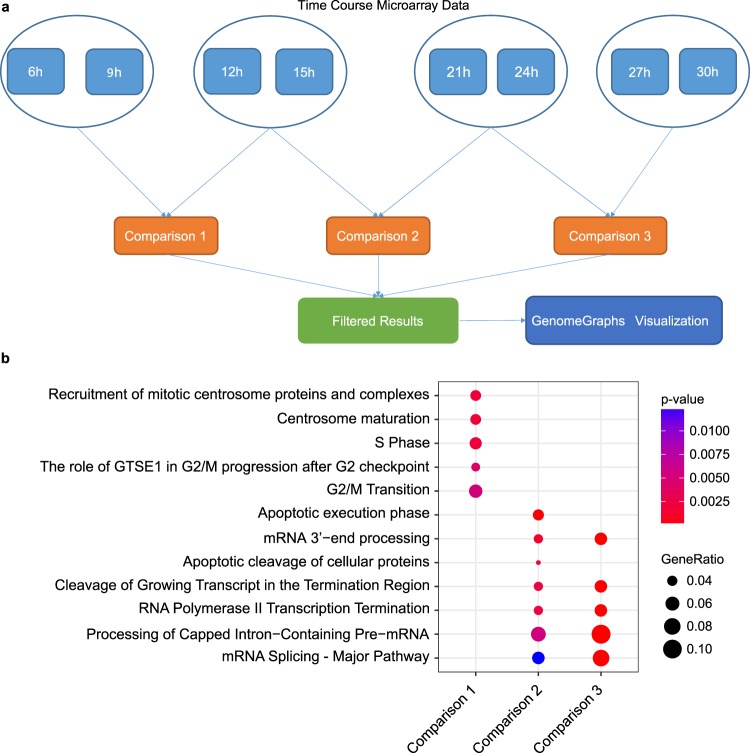
Numerous alternative splicing events were detected in the time-course data for the HD-MY-Z cell line. (**a**) Experimental design for the grouping and subsequent analysis of the time-course microarray data with the EventPointer R package. The time points were grouped in four sets that resulted in three EventPointer comparisons. The data obtained with EventPointer was additionally filtered for *p* < 0.01 and absolute delta-PSI > 0.1. The selected genes were visualized at the probe set level with the GenomeGraphs package. (**b**) Enrichment analysis for Reactome pathways for the genes detected to be alternatively spliced in the three comparisons. The gene ratio denotes the ratio between the number of the genes associated with the term and the total number of genes associated with the term in the pathway.

Given the seemingly circadian regulation of SF, we set up to investigate whether the interplay between the clock and the SF elements had an impact on splicing decisions detectable in HD-MY-Z cell lines. We used a recently published time-course data set from our group on the Hodgkin lymphoma cell line HD-MY-Z (ArrayExpress E-MTAB-6400^27^) to investigate differential splicing events. This data set comprises of 9 samples per cell line, for the cell lines HD-MY-Z and the lymphoblastoid B cell line LCL-HO (RNA collected between 6 h and 30 h after synchronization, in 3 h intervals). We took the splicing-sensitive Affymetrix HTA-2.0 arrays as a platform for the generation of the time-course expression profiles, and analysed the data with the EventPointer R^68^ package as described in Materials and Methods. The package supports the inclusion of junction probe information in the analysis, which other algorithms focusing on exon arrays lack. We clustered the samples from the individual time points with hierarchical clustering to determine similar-behaving time points for the subsequent EventPointer comparisons. We excluded the 18 h time point based on the PCA and hierarchical clustering analysis provided in Supplementary Fig. S6. The time point was excluded from the subsequent search for splicing events due to the large distance of the data from the other time points, which might affect the results in the needed pairwise analysis. We grouped the time points in bins containing two time points in chronological order (e.g. 6 h and 9 h compared to 12 h and 15 h). The process of binning of time points is widely used to increase the signal to noise ratio^69–72^. This allowed us to produce three comparisons for the subsequent analysis with EventPointer (Fig. 4a).We obtained a list of genes for which alternative splicing is detected for each comparison (Supp. Tables S6–S8). Moreover, we determined the type of event detected, its position, the associated *p*-value and the delta-PSI (delta percent spliced in index) (Comparison 1 – Supp. Table S6, Comparison 2 **–** Supp. Table S7, Comparison 3 – Supp. Table S8). The percent spliced-in index (PSI) denotes the ratio between probe sets including or excluding the exons, and thus provides information concerning the degree of variation in splicing^68^. We filtered the resulting data for events with *p* < 0.01 and absolute delta-PSI > 0.1, to identify significant differential splicing events across the time points. The PSI filtering identifies the changes in alternative splicing that shift the balance of isoforms and hence have the highest potential for biological impact. The delta-PSI values showing this behaviour after *p*-value filtering are depicted in Supplementary Fig. S7. In comparison 1, we detected 249 (Supp. Table S6) unique genes to be alternatively spliced after filtering. In comparison 2, 261 (Supp. Table S7) unique genes are alternatively spliced after filtering, and in comparison 3 we found 235 unique genes (Supp. Table S8) alternatively spliced. We further investigated this gene set by creating a comparative analysis for Reactome terms enriched in the different time points (Fig. 4b, Supp. Table S10). The gene set in comparison 1 shows an association to the cell cycle (G2/M Transition, *p* = 5.271E-03, Mitotic G1-G1/S phases, *p* = 4.430E-03). The gene sets in comparison 2 and 3 differ mainly in the strong signal of the apoptotic execution phase present in comparison 2. This enrichment in comparison 2 (*p* = 6.790E-05) is caused by the genes *ADD1* (adducin 1), *SPTAN1* (spectrin alpha, non-erythrocytic 1), *ACIN1* (apoptotic chromatin condensation inducer 1) and *GSN* (gelsolin), all targets of the caspase 3 and elements of the apoptotic execution pathway. Both comparisons 2 and 3 contain enrichments of terms related to mRNA processing and splicing (Processing of Capped Intron-Containing Pre-mRNA, *p* = 3.373E-06), highlighting the potential circadian regulation of such processes.

Following the application of the previously described filtering, we obtained 623 unique genes after merging the data from comparison 1, comparison 2 and comparison 3. These genes represent the ones that passed filtering and for which an alternative splicing event or events was detected in at least one comparison for the HD-MY-Z cell line. Additionally, we performed a correlation analysis between the expressions of oscillating SFs and AS genes in the HD-MY-Z cell lines (Supp. Fig. S5), we observe a strong and widespread positive correlation between the two data sets, indicating an important connection between the SFs and the AS genes. Our results identified a set of alternatively spliced genes in HD-MY-Z cells, which are involved in key cellular pathways that affect apoptosis and the cell cycle and consequently are likely to play a role in cell proliferation.

### Circadian regulation of splicing factors leads to switches in alternative splicing of target genes in HL cell lines

Furthermore, we searched for a temporal pattern in the detected splicing events. To investigate the existence of potential temporal changes in the spliced genes, possibly in a rhythmic manner, we extracted the genes that undergo alternative splicing events in each of the three comparisons for the HD-MY-Z cell line at the same genomic coordinates according to the annotation and with a p-value cutoff of 0.01 and absolute delta-PSI value of at least 0.1 in at least one comparison, as described in the previous section. Our analysis resulted in a list of 42 alternatively spliced genes for which alternative splicing events are present in all comparisons with *p* < 0.01 (Supp. Table S9) and show the highest variance in delta PSI in the three comparisons. The variance analysis was performed to focus on genes with potential changes in splicing over time. Indeed, out of the 42 genes 21 show an oscillatory behaviour of their respective gene expression as detected by MetaCycle. We ordered the genes varying in each comparison by the variance in their delta-PSI values, which reflects strong changes in splicing between the comparisons. Several genes within this set, such as *DPYD* (*p* = 3.17E-04, Supp. Table S8) and *POP4* (*p* = 4.69E-05, Supp. Table S8), show temporal changes between the expressions of one or more exons in the HD-MY-Z cell line. EventPointer classifies this event as a “Cassete Exon”, a case of exon skipping. Both *DPYD* and *POP4*, involved in drug metabolism and RNA processing/transport, respectively, oscillate in a circadian manner as detected in our analysis with MetaCycle. Other interesting candidates are *MAGOH* (*p* = 3.90E-06, Supp. Table S8) involved in RNA transport, and *SS18* (Synovial Sarcoma Translocation, Chromosome 18), *p* = 3.75E-07 (Comparison 3, Cassette Exon type splicing event), which is related to synovial sarcoma and its splice variant lacking exon 8 was shown to increase the malignant potential in synovial sarcoma^73^ (Supp. Table S8). A further cancer-related gene with rhythmic AS, based on our results, is *VIPR1* (Vasoactive Intestinal Peptide Receptor 1 gene, *p* = 9.71E-05, Supp. Table S8). A recent study comprehensively investigated the cytoprotective effects of the small neuropeptide VIP and its receptor (VIPR1) in cancer stem cells and identified an antiapoptotic function of the VIP receptor in these cancer cells^74^.

**Figure 5:**
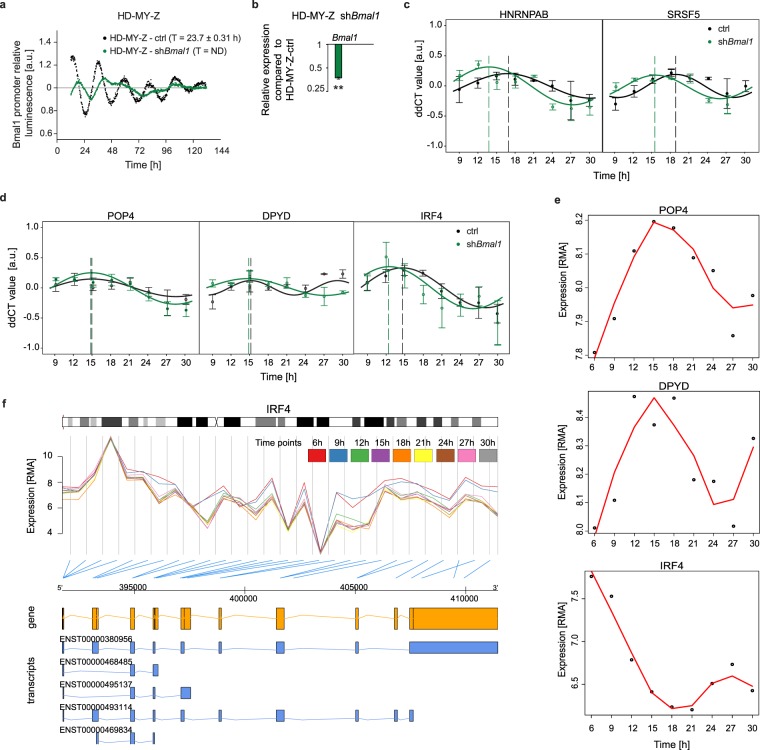
The knockdown of the core-clock gene *Bmal1* affects the expression of SF and alternatively spliced genes. (**a**) Bioluminescence measurements of *Bmal1* promoter activity in HD-MY-Z control (ctrl) cells and HD-MY-Z-shBmal1 cell lines. Bioluminescence was measured for approximately five consecutive days. Shown is one representative replicate and the mean value for the period (T) evaluated with Chronostar, ND = non-defined. Mean ± SEM, n = 3. (**b**) Gene expression analysis of *Bmal1* after sh*Bmal1* compared to the corresponding control gene. Mean ± SEM, n = 3. Knockdown efficiency in HD-MY-Z-shBmal1 cells, 0.370 ± 0.009 (63%), **p < 0.01. (**c**) Harmonic regression of RT-PCR data for splicing genes (*HNRNPAB*, *SRSF5*) and for (**d**) alternatively spliced genes (*POP4*, *DPYD*, and *IRF4*) in the control (black) and sh*Bmal1* (green) conditions for the cell line HD-MY-Z. The solid lines show the best fitting harmonic regression and the dashed vertical lines show the phase of the oscillation (p < 0.05, mean ± SEM, n = 3; n = 2, *DPYD* time point 27 h). (**e**) The expression levels of *IRF4*, *DPYD* and *POP4* shows rhythmic behaviour (analysis with MetaCycle). (**f**) Visualization of the expression data from all nine time-course arrays for the HD-MY-Z cell line for *IRF4*. The structure of the gene and the corresponding known isoforms are displayed. *IRF4* was detected to be alternatively spliced with the event type - alternative last exon. In this case, two time points (6 h, 9 h) present a significantly different isoform balance then the remaining time points. Of the known transcripts, only the longer transcripts ENST00000380956 and ENST00000493114, which seem to be the ones affected by the change in balance, are known to result in protein products.

To further investigate the putative connection between the circadian clock and rhythmically expressed SFs and AS genes, we generated a shRNA knockdown cell line of the core-clock gene *Bmal1* (HD-MY-Z-sh*Bmal1*) and evaluated the knockdown efficiency using a bioluminescence assay for *Bmal1* promoter activity recordings (Fig. 5a,b and Supp. Fig. S12). As seen from our data, the expression pattern of *Bmal1* promoter activity is reduced in the sh*Bmal1* cell line as compared to the control (HD-MY-Z-ctrl, transduced with an empty vector). Next, we generated a time-course data set (9h-30h, in 3 h intervals) for both the control and sh*Bmal1* cell lines and measured gene expression levels for two SFs (*SRSF5* and *HNRNPAB*, Fig. 5c) and three AS genes (*IRF4*, *DPYD* and *POP4*, Fig. 5d) by RT-PCR. From the harmonic regression analysis, we observed that all genes analysed show rhythmic expression (*p* < 0.05), with phases similar to the ones identified via our bioinformatic approach (Figs 3 and 5e). The best fitting curves are depicted in Fig. 5c,d and their circadian parameters (period, p-value, amplitude, acrophase, and acrophase shift between control and shBmal1 condition) are provided in Supplementary Table S11 for variable periods and Supplementary Table S12 for 24h-periods. Furthermore, we identified different oscillation phenotypes in the sh*Bmal1* cell line as compared to the control cell line, reinforcing our results on a possible link between the circadian clock and rhythmically expressed SFs and AS genes (Fig. 5c,d). We observed that the perturbation in the clock gene *Bmal1* affected the expression of SF and alternatively spliced genes. In the sh*Bmal1* condition, the phase of *POP4*, *DPYD*, and *IRF4* genes occurred 0.12 h, 0.51 h, and 2.17 h before their corresponding peaks in the control condition, respectively (Supp. Table S11). As expected, the differences in the oscillatory expression phenotype between both conditions were more pronounced for the SF genes. In sh*Bmal1* condition, the *HNRNPAB* and *SRSF5* had a phase of expression 2.99 h and 3.30 h before their corresponding expression peaks in the control condition, respectively (Supp. Table S11). These results point to a potentially closer and more direct relation between SF and clock genes, as compared to the clock genes and the alternatively spliced candidate genes.

While investigating the previously mentioned list of 42 genes (alternatively spliced genes for which alternative splicing events are present in all comparisons, Supp. Table S9) that undergo alternative splicing and hence show high delta-PSI variance in the HD-MY-Z cell line, we detected a strong signal for *IRF4* (*p* = 3.29E-05, Supp. Table S8) from an event classified as an “alternative last exon”. A strong difference between the signals from the 6 h and 9 h time points as compared to the remaining time points is visible (Fig. 5f). *IRF4* shows oscillations in expression (*p* = 7.90E-05, Fig. 5e), and the alternative splicing signal for *IRF4* is present in each comparison. *IRF4* has five known isoforms, for which however only two lead to known proteins, the ENST00000380956 and the ENST00000493114. The short isoform, ENST00000493114, does not include the last exon (ENSE00001957207) of the gene, which can account for the signal detected. This isoform also differs from the canonical ENST00000380956 form by not fully including the interferon regulatory factor 3 domain and the SMAD/FHA domain, otherwise present (Fig. S10). Most importantly, this isoform undergoes nonsense-mediated decay (NMD). Interestingly, the *IRF4* gene has a high expression in Hodgkin Lymphoma (observed in stage IV HL cell lines), as well as an anti-proliferative and pro-apoptotic effects in several HL cell lines upon silencing^75,76^. To verify if the observed splicing behaviour differs from a non-cancer cell line, we analysed a 24 h time-course data set for the LCL-HO cell line (B lymphoblastoid cell line). In this cell line the difference in *IRF4* splicing was not observed (Supp. Fig. S8). Additionally, the genes *CABYR* (calcium binding tyrosine phosphorylation regulated), *ARHGEF39* (rho guanine nucleotide exchange factor 39), *NUDT16* (nucleoside diphosphate-linked moiety X motif 16), *SEPT10* (septin 10), *CDC45* (cell division cycle 45) and *TUBA3C* (tubulin alpha 3c), for which we also detected significant changes in the splicing over time for the HD-MY-Z cell line are shown in Supplementary Fig. S9. Interestingly, while some of the time-specific alternative splicing events are present in both cell lines with similar patterns in the expression of the probesets, others show strong differences such as large changes of expression over time that only affect a part of the probes of a gene and hence point at alternative splicing changes over time that differ between the cell lines. This is particularly visible for *CABYR*, the splicing of this gene varies over time for the LCL-HO and HD-MY-Z cell lines. Different splice variants of *CABYR* have been identified so far, which show different calcium-binding capacities^77^ and the differential expression of these variants was shown to exhibit aberrant expression in brain tumours and brain cancerous cell lines^78^.

**Figure 6:**
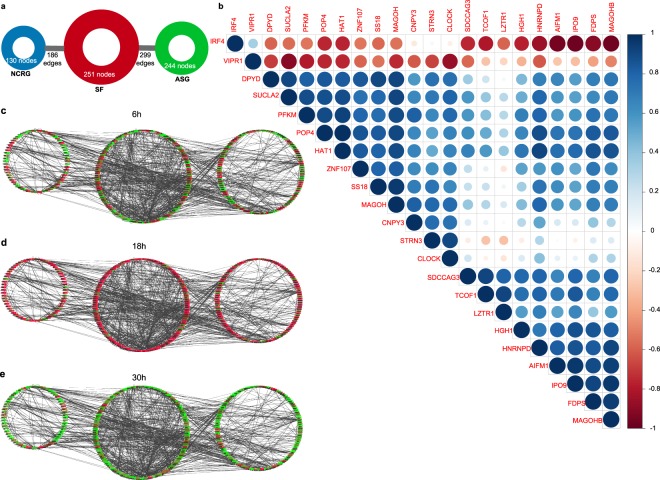
The SF and the NCRG interact with the alternatively spliced genes and show similar rhythms in the HD-MY-Z cell line. (**a**) The previous NCRG interacting with the SF was extended to include the alternatively spliced genes (ASG) for the HD-MY-Z cell line. This new network shows 299 direct interactions between the alternatively spliced genes and SF and another 222 direct interactions between the alternatively spliced genes and the NCRG. (**b**) Correlation analysis of 21 of the 42 genes for which changes in alternative splicing were detected in each comparison of the HD-MY-Z time course and the CLOCK gene. The expression of the 21 genes oscillates with approximately circadian rhythm according to analysis performed with MetaCycle. The expression of multiple genes such as POP4 correlates with the expression of CLOCK. Binding sites for CLOCK were detected in the promoter regions of the splicing factors (*p* = 2.60E-25, Fig. 2b). The expression of multiple genes that undergo splicing over time and oscillate with approximately circadian rhythm is correlated with the expression of *CLOCK*: *VIPR1* (correlation. −0.86), *DPYD* (0.76), *POP4* (0.68), *STRN3* (0.90). Red colour denotes negative correlation and blue colour denotes positive correlation with the size and intensity of the circles reflecting the difference of the value from 0. (**c**) Network of the splicing factors and circadian regulated genes connected to the alternatively spliced genes. Expression data from the time-course HTA 2.0 microarrays was added to the network. The new network is reduced to the genes for which expression data could be obtained from the arrays and contains a total of 290 nodes and 409 edges. Of those, 62 nodes are part of the NCRG, 76 nodes are part of the SF, 132 nodes are part of the spliced genes and 16 nodes belong to both the SF and the NCRG underlining their close interaction. The expression values shown are for the 6 h time point and normalized in the range (−1, 1) with red representing high expression and green representing low expression. (**d**) Identical network structure with the expression data for the 18 h time point and normalized in the range (−1, 1) with red representing high expression and green representing low expression. The expression peaks at this time point as also visible in the heatmaps in Fig. 2 (**e**) Identical network structure with the expression data for the 30 h time point and normalized in the range (−1,1) with red representing high expression and green representing low expression. Following the peak at 18 h the 30 h time point shows a strong reduction in expression.

To explore the role of the circadian clock as a regulator of the observed alternative splicing events, we further analysed the interactions between the identified alternatively spliced genes, the NCRG and the SF genes sets. For this, we expanded the previously established NCRG by including the SF and the 623 alternatively spliced genes identified by our pipeline (unique genes in all comparisons) in the HD-MY-Z cell line, regardless of their circadian behaviour. We also included all interactions retrieved from the IntAct database (Fig. 6a). This new network is based on the previous network of the NCRG and splicing factors (Supp. Fig. S2) and contains 299 direct interactions between the alternatively spliced genes and SF, 222 direct interactions between the alternatively spliced genes and the NCRG as well as 186 connections between the splicing factors and the NCRG. A detailed view of the network is provided in Supplementary Fig. S11. To further explore the network, we integrated the expression data from the time-course arrays for the HD-MY-Z cell line. The three groups showed similar oscillation pattern and an overall change towards higher expression levels at 18 h (Fig. 6c–e). Altogether, our data point towards a correlation between the circadian clock phenotype and the expression patterns of SF in HD-MY-Z cells. This is further shown by the correlation between the expression of the gene *CLOCK* with the expression of several of the genes undergoing changes in splicing over time (Fig. 6b). We found enriched binding sites for the *CLOCK* gene in the promoter regions of the SF (*p* = 2.60e-25), if the splicing factors are putatively regulated by the clock their subsequent output in AS is likely to be regulated as well. We investigated this hypothesis on the expression of the 21 genes previously detected to undergo changes in splicing in each comparison and having oscillations with a period of 24 ± 3 h (*p* < 0.05 as calculated by MetaCycle). The previously detected genes with splicing events correlate (Pearson correlation) with the expression of *CLOCK* – *VIPR1* (correlation, −0.86), *DPYD* (correlation, 0.76), *POP4* (correlation, 0.68), *STRN3* (correlation, 0.90). This interplay between the clock network and the SFs results in temporal changes of AS in these cells and suggests a regulatory role of the clock as a possible effector of cancer phenotype via the splicing machinery.

## Discussion

In this study, we explored the role of the circadian clock network in influencing alternative splicing in HD-MY-Z cells with a bioinformatics approach using time-course microarray data recently published by our group^27^. We built upon previously published data, which reported stage-specific patterns of AS and SF for HL cells from different tumour stages^11^. These patterns were successfully used to classify the stage of each tumour and to distinguish between a non-tumour cell line and cancer^11^. To explore the role of the circadian clock as an important and mediatory partner in this interplay, we first determined the number of oscillating genes in the NCRG and SF groups in several time course array data sets for mammalian tissues and cell lines, and compared their expression and oscillatory patterns (Supp. Figs S3 and S4). We observed a correlation between the number of oscillating genes in NCRG and SF with similar rhythmic expression patterns across various mouse tissues, and in human cancer cell lines (U2OS and HD-MY-Z). It is important to mention, that the oscillation profile of gene expression within different cancer cell lines, as well as compared to a non-cancer cell line (LCL-HO) is different. Previous studies have shown, that various cancer cell lines exhibit a range of diverse oscillation phenotypes^28^. In a recent study by our group, we examined the oscillation pattern of gene expression in the HL cell line HD-MY-Z compared to a lymphoblastoid B cell line LCL-HO (as a non-cancer cell model) and found a strong connection between the oscillating genes and cancer related pathways, in the HD-MY-Z cell line^27^. Thus, in this particular cellular model, a properly functional circadian clock in the HD-MY-Z cell line might be relevant for the malignant phenotype of these cancer cells. As seen from the time-course array data for several mouse tissues and human cell lines (summarized in Fig. 2 and Supp. Fig. S3), these expression patterns and correlations are tissue-specific, pointing to the distinct and specific circadian regulation of physiological and cellular processes in each tissue and cell type. Interestingly, we showed that several core-clock and clock-related genes (*AHR*, *ATF2*, *CLOCK*, *SMAD4*, *TEF*, *IRF7*, *SP1*, *ARNTL*, *ARNT2 and EGR1*) recognize the promotor region of SF and act as TFs directly regulating the expression of the elements of the splicing machinery (Fig. 2a,b).

Previous studies, including work from our group indicate that the circadian clock plays a key role in regulating metabolism^26^, the cell cycle and cell proliferation^25^. Given the interplay between the clock and SF highlighted by our data, we hypothesise that one possible additional way via which the clock affects cellular processes, including the cell cycle, is by the temporal regulation of splicing events of the effector genes. Indeed, the observed temporal change in the expression of SFs correlates with changes in alternative splicing, which occur in the cancer cell line and that influence apoptosis and proliferation. Using our bioinformatics pipeline applied to multiple comparisons of time points, we investigated the impact of the differential oscillations of SFs in alternative splicing, and identified several alternatively spliced genes in the HD-MY-Z cell line with rhythmic patterns of isoform expression. One example is the *SS18* gene involved in transcriptional dysregulation in cancer which is associated with synovial sarcoma^79^. Another gene with rhythmic alternative splicing is *MAGOH* involved in RNA transport, splicing and nonsense-mediated decay. We further identified several genes involved in metabolism, among them the *DPYD*, involved in drug metabolism. *DPYD* is associated with dihydropyrimidine dehydrogenase deficiency, leading to increased risk of toxicity in cancer patients receiving chemotherapy^80^. Effectors of the cell cycle are differentially spliced during a 24 h period in our data sets. In particular, *VIPR1*, which is involved in the antiapoptotic pathway in cancer cells and acts as proliferation regulator^74^. These results highlight the significance of a failure in the temporal control of alternative splicing which influences major cellular pathways with possible subsequent effects in cancer onset or progression. One of the genes that we identified to be alternatively spliced in a timely manner in HD-MY-Z was *IRF4* (Fig. 5f). This gene is a member of the IRF family of transcription factors expressed in cells of the immune system. IRF4 acts as a key regulator of several steps in lymphoid and myeloid cell differentiation, and is associated with many lymphoid malignancies. Previous studies showed that *IRF4* is highly expressed in HL cells and its silencing inhibits HL cell proliferation and induces apoptosis^76^. Our data suggest that the rhythmic alternative splicing of *IRF4* may contribute to its over expression in HD-MY-Z, which in turn may regulate the progression stage of tumorigenesis in these cells. Since this rhythmic alternative splicing is directly correlated with the clock, we hypothesise that changes in clock expression are correlated with the AS pattern of *IRF4*, hence affecting the cancer phenotype. Our hypothesis is further supported through the RT-PCR data, where we identified oscillations in the expression of the SFs *HNRNPAB* and *SRSF5*, as well as in the alternatively spliced candidate genes *IRF4*, *POP4* and *DPYD* in the control condition and a different oscillatory phenotype for the same genes in the *Bmal1* knockdown cell line (Fig. 5c,d). The fits obtained from the harmonic regression analysis are statistically significant (p-value < 0.05), and the circadian parameters derived from the analysis (e.g.: acrophase, period, and amplitude) differ between control and sh*Bmal1* condition (Suppl. Table S11, Suppl. Table 12). Yet, to fully evaluate these results and find possible mechanistic explanations at both molecular and cellular levels, further experimental studies need to be performed in future follow up studies also at the protein level. Of note, it was previously reported that though no, or weak, rhythmic gene expression at the transcript level was detectable, the protein level showed robust rhythmic oscillation. Gotoh and colleagues showed strong rhythmic protein levels of p53 although there was no oscillation detectable at the mRNA level^81^.

Altogether, our data points toward an additional role of the circadian clock in controlling RNA processing via the temporal regulation of the splicing machinery. Given the impact of this regulation in alternative splicing and the known relevance of the latest in cancer progression, our data highlights a layer of temporal transcriptional complexity likely to have an impact in tumorigenesis.

## Materials and Methods

### Cell culture

HD-MY-Z (DSMZ – #ACC 346) cells were maintained in RPMI 1640 (Gibco) supplemented with 1% penicillin-streptomycin (Gibco) and 10% foetal bovine serum (Gibco). Stable-transduced cells were selected and maintained in medium containing 100 μg/mL hygromicin B (Gibco) for the Bmal1:luc hygromicin (BLH) and 10 μg/mL of puromycin for the shRNA KD of the clock gene *BMAL1*. For live-cell bioluminescence recording (evaluation/analysis/monitoring), cells were maintained in phenol red-free RPMI 1640 (Gibco) containing 10% foetal bovine serum, 1% penicillin-streptomycin and 0.1 mM D-Luciferin (PJK). All cells were incubated at 37 °C with 5% CO2 atmosphere.

### Lentivirus production

Lentiviral elements containing a BMAL1-promoter-driven luciferase (BLH) and an empty vector (TRC Lentiviral pLKO.1 Empty Vector Control Dharmacon Inc., Lafayette, CO) or the shRNA KD (TRC Lentiviral Human ARNTL shRNA - Clone ID: TRCN0000019097) were used in the present work. HEK293T (human, kidney, ATCC Number: CRL-11268) cells were seeded in 175 cm2 culture flasks and co-transfected with 12.5 µg packaging plasmid psPAX, 7.5 µg envelope plasmid pMD2G and 17.5 µg BLH or shRNA KD expression plasmid using the CalPhos mammalian transfection kit (Clontech) according to the manufacturer’s instruction. To harvest the lentiviral particles, the supernatant was centrifuged at 4100 × g for 15 min to remove cell debris and passed through a 45 µm filter. Lentivirus were stored at −80 °C.

### Transduction with lentiviral vectors

1 × 10^6^ cells were seeded in a 6-well plate with 1 mL of RPMI1640, 10% foetal bovine serum, 1% penicillin-streptomycin and 4 µg/mL Polybrene (Sigma). 1 mL of supernatant of the corresponding lentivirus were added to each well and plates were centrifuged for 90 min at 34 °C at 1000 g. The supernatant was discarded after 4 hours incubation at 37 °C with 5% CO2 and 2 mL of fresh medium was added. Two days after, the medium was replaced by selection medium (supplemented with hygromicin S or puromycin (according to the lentivirus construct used)) and incubated at 37 °C with 5% CO2 atmosphere. Cells were maintained in selection medium for at least two weeks. For the shRNA KD, the KD efficiency was determined by gene expression analysis.

### Synchronization and measurement of circadian rhythms

For bioluminescence measurement, 0.5–1 × 10^6^ cells were washed once with 1 × PBS, plated in 35 mm dishes and synchronized by adding fresh phenol-red-free RPMI 1640 medium supplemented with 10% foetal bovine serum, 1% penicillin-streptomycin and 0.1 mM D-Luciferin. Bmal1-promoter-(BLH)-reporter activity was measured, using the LumiCycle instrument (Actimetrics). After bioluminescence recording for 5–7 days, phase, period and amplitude were analysed with the ChronoStar^82^ analysis software V3.0 using 24 h running average de-trended method and fitting cosines curves were calculated as output.

### RNA isolation and cDNA synthesis

Cells were seeded in 35 mm dishes with a density of 5 × 105 cells/dish and synchronized by fresh medium addition. Cells were collected every three hours, in triplicates and the pellets were stored at −80 °C. Total RNA was isolated using the RNeasy extraction kit (Qiagen), including DNase digestion, according to the manufacturer’s instructions. Cells were lysed with 350 µl RLT buffer and the lysate was homogenized. Total RNA concentration was determined using NanoDrop® ND-1000 UV-Vis Spectrophotometer and stored at −80 °C °C. 1 µg of total RNA was reverse-transcribed to cDNA with M-MLV reverse transcriptase (Invitrogen), random hexamers (Eurofins MWG Operon) and dNTPs Mix (Thermo Fisher).

### Quantitative real-time PCR

For gene expression quantification, we used SYBR-Green (Bio-Rad Laboratories, Hercules, CA) based real-time PCR in 96-well plates and the human commercial primers from QuantiTect primer assays (Qiagen) for *GAPDH* and *BMAL1*. For *POP4*, *DPYD*, *IRF4*, *HNRNPAB*, *and SRSF5* genes following primers were used: *POP4* (Forward: 5′CGG TCC GAG AAT GAA GAG TGT; Reverse: 5′GGC TGG ACA TCG GAG TCA TT); *DPYD* (Forward: 5′ACT CTG TGT TCC ACT TCG GC; Reverse: 5′CAG GCA TCT CAT TGC TTC TCG); *IRF4* (Forward: 5′GTG AAA ATG GTT GCC AGG TGA; Reverse: 5′AGG CTT CGG CAG ACC TTA TG); *HNRNPAB* (Forward: 5′TGT CAG TGG AAG CAA GTG TGA; Reverse: 5′CTG GTT CCA GTA GTT GCC GT); *SRSF5* (Forward: 5′CAG CAA AAG GCA CAG TAG GT; Reverse: 5′CCG GCT GTA AGA TTT GCG AC). The reaction was performed in a real-time PCR Detection System (Bio-Rad). Each sample was measured in triplicates and the CT values were determined by using the regression method. The expression levels were normalized to those of *GAPDH* (ΔCT) and calibrated to the mean expression value of each gene (time-course analysis) or in relation to the control (ΔΔCT). The relative quantification was performed using the 2−ΔΔCT method^83^.

### Western blotting

For protein isolation, cells were gently detached from the dish, sedimented by low-speed centrifugation and resuspended in lysis buffer. Aliquots containing 30 µg of proteins from each cell lysate were used for SDS polyacrylamide gel electrophoresis and transferred to a nitrocellulose membrane (GE Healthcare) using the Trans-Blot Turbo Transfer System (Bio-Rad). Membranes were probed with the following primary antibodies: BMAL1 (1:2000; Abcam ab93806) and GAPDH (1:2500; Abcam ab9485). After incubation with corresponding suited horseradish peroxidase-conjugated secondary antibody (1:2000; Abcam ab205718), signals were developed using the enhanced chemiluminescence kit (ECL™ Prime Western Blotting System, GE Healthcare), acquired by ChemiDoc Imaging System XRS + (Bio-Rad) and analysed for densitometry with the ImageJ software.

### Cancer cell lines and sampling

HD-MY-Z (DSMZ – #ACC 346: nodular sclerosing Hodgkin lymphoma stage IIIb refractory to therapy) and LCL-HO (DSMZ – #ACC 185: B lymphoblastoid cells) this data set was recently published in Abreu *et al*.^27^, cells were synchronized by medium change and samples were collected every three hours between ZT06h and ZT30h. One array per time point was hybridized at the LFGC (Labor für funktionelle Genomforschung, Charité - Universitätsmedizin Berlin). The microarray dataset is deposited in the ArrayExpress database at EMBL-EBI (www.ebi.ac.uk/arrayexpress) under the accession number E-MTAB-6400^27^.

### Enrichment analysis

Enrichment analysis was performed with the clusterProfiler^84^ and ReactomePA^85^ R packages using Reactome Pathways. The enrichment of Reactome pathways was performed with a p-value cutoff of 0.05, q-value cutoff of 0.2, “universe” selection for background genes and a Bonferoni-Hochberg correction for multiple testing. ClusterProfiler analysis was performed with a p-value cut-off of 0.05 and no correction for multiple testing. Enrichment analysis for transcription factor binding sites was performed with the MEME suite’s^86^ AME tool and the HOCOMOCO^47^ database v10.

### Network creation and random network analysis

The protein-protein interaction networks were created with data from the IntAct database. Cytoscape 3.6^87^ was used for visualization and network clustering with the clusterMaker app. For the generation of random networks we used the 2015 IntAct snapshot present in the iRefIndex^36^ database and the iRefR R^88^ package. Network clustering was performed with the clusterMaker^89^ Cytoscape app.

### Array processing and detection of oscillating genes

The time-course data from the Affymetrix HTA-2.0 arrays were analysed with R and the oligo package, the normalization was performed with the RMA algorithm on the TCO level for oscillating gene analysis. On TCO level the arrays were annotated with the hta20transcriptcluster.db package from Bioconductor^90^. The computational analysis for oscillating genes was performed with the MetaCycle^59^ R package with all supported methods (ARS, JTK, LS) for the period range of 21 h to 27 h. The reason for selecting this period range is based on a recent published study by our group investigating the oscillation of gene expression in the mentioned cell lines^27^. Only oscillating genes with a combined p-value lower than 0.05 were used for the further analysis. The microarray dataset was deposited in the ArrayExpress database at EMBL-EBI (www.ebi.ac.uk/arrayexpress) under the accession number E-MTAB-6400 and will be release d upon publication. The mouse arrays were retrieved from circadb (circadb.hogeneschlab.org)^91^.

### Detection and visualization of splicing events

For the detection of splicing events the arrays were pre-processed with the aroma.affymetrix R package and analysed with the EventPointer R^68^ package with the annotation cdf suggested by the package author. Each two time points were taken as replicates and three pairwise comparisons were so performed for each cell line. The 18 h time point was excluded based on clustering data and PCA given in Supplementary Fig. S5. The detected candidate genes were visualized with GenomeGraphs^92^ R package from data on probe set level passed through the RMA algorithm. Genomic locations of the probe sets were retrieved from the Affymetrix annotation and data on the isoforms and the gene structure was retrieved from ensembl biomart grch37. Probe set level annotation was provided by the hta20probeset.db package in Bioconductor^90^.

### Statistical analysis

The experiments were carried out with three biological replicates for each condition (for time point 27 h of *DPYD*, n = 2). All results are represented as mean ± SEM. Statistical analysis was performed using two-tailed unpaired t-test. A *p*-value < 0.05 was considered as statistical significant (*p < 0.05; **p < 0.01; ***p < 0.001). The rhythmicity of the genes *HNRNPAB*, *SRSF5*, *POP4*, *DPYD*, and *IRF4* (control and sh*Bmal1* condition) was tested by fitting a linear sine-cosine function to the time-course data (ΔΔCT normalised to the mean) using the R package HarmonicRegression^93^. Eight time points (9 h–30 h) were included in the analysis. The harmonic regression procedure fits the model y(t) = m + acos(ωt) + bsin(ωt) to the time-course data to estimate absolute amplitudes (A = √(a^2^ + b^2^)) and phases (ϕ = atan2(b,a)) for a given period^93^. Periods from 14 h to 27 h in increments of 0.1 h were tested. The circadian parameters (*p*-value, amplitude, acrophase), as well as the acrophase shift between control and shBmal1 condition were determined. The significance was calculated with an F-test of the model fit and bounded by *p* < 0.05. The best fitting curve (lowest *p*-value) was used for visualization.

### Data access

The microarray data For the HD-MY-Z and LCL-HO cell lines from this study is deposited in the ArrayExpress database at EMBL-EBI (www.ebi.ac.uk/arrayexpress) under the accession number E-MTAB-6400. The mouse arrays were retrieved from circadb (circadb.hogeneschlab.org)^91^ under the GEO identifiers: GSE11923 (Mouse Liver), GSE25585 (Mouse Macrophages), GSE11922 (NIH 3T3) or directly from circadb (Mouse Pituitary^58^). The additional U2OS arrays can be found under the GEO identifier GSE13949.

## Supplementary information

Supplementary Information

Fugure S1

Figure S2

Figure S11

Table S1

Table S2

Table S3

Table S4

Table S5

Table S6

Table S7

Table S8

Table S9

Table S10

## References

[1] Wahl MC, Will CL, Luhrmann R (2009). The spliceosome: design principles of a dynamic RNP machine. Cell.

[2] Wang ET (2008). Alternative isoform regulation in human tissue transcriptomes. Nature.

[3] Kelemen O (2013). Function of alternative splicing. Gene.

[4] Chen M, Manley JL (2009). Mechanisms of alternative splicing regulation: insights from molecular and genomics approaches. Nat Rev Mol Cell Biol.

[5] Venables JP (2004). Aberrant and alternative splicing in cancer. Cancer Res.

[6] Venables JP (2009). Cancer-associated regulation of alternative splicing. Nat Struct Mol Biol.

[7] Auboeuf D, Carmo-Fonseca M, Valcarcel J, Biamonti G (2012). Alternative splicing and cancer. J Nucleic Acids.

[8] Ghigna C, Valacca C, Biamonti G (2008). Alternative splicing and tumor progression. Curr Genomics.

[9] Xu Q, Lee C (2003). Discovery of novel splice forms and functional analysis of cancer-specific alternative splicing in human expressed sequences. Nucleic Acids Res.

[10] Hui L (2004). Identification of alternatively spliced mRNA variants related to cancers by genome-wide ESTs alignment. Oncogene.

[11] Relogio A (2005). Alternative splicing microarrays reveal functional expression of neuron-specific regulators in Hodgkin lymphoma cells. J Biol Chem.

[12] Karni R (2007). The gene encoding the splicing factor SF2/ASF is a proto-oncogene. Nat Struct Mol Biol.

[13] Thorsen K (2008). Alternative splicing in colon, bladder, and prostate cancer identified by exon array analysis. Mol Cell Proteomics.

[14] Golan-Gerstl R (2011). Splicing factor hnRNP A2/B1 regulates tumor suppressor gene splicing and is an oncogenic driver in glioblastoma. Cancer Res.

[15] Guo X, Chen QR, Song YK, Wei JS, Khan J (2011). Exon array analysis reveals neuroblastoma tumors have distinct alternative splicing patterns according to stage and MYCN amplification status. BMC Med Genomics.

[16] Cohen-Eliav M (2013). The splicing factor SRSF6 is amplified and is an oncoprotein in lung and colon cancers. J Pathol.

[17] McGlincy NJ (2012). Regulation of alternative splicing by the circadian clock and food related cues. Genome Biol.

[18] Preussner M (2014). Rhythmic U2af26 alternative splicing controls PERIOD1 stability and the circadian clock in mice. Mol Cell.

[19] Kojima S (2007). LARK activates posttranscriptional expression of an essential mammalian clock protein, PERIOD1. Proc Natl Acad Sci USA.

[20] Lee KH (2012). Rhythmic interaction between Period1 mRNA and hnRNP Q leads to circadian time-dependent translation. Mol Cell Biol.

[21] Morf J (2012). Cold-inducible RNA-binding protein modulates circadian gene expression posttranscriptionally. Science.

[22] Cao R (2013). Translational control of entrainment and synchrony of the suprachiasmatic circadian clock by mTOR/4E-BP1 signaling. Neuron.

[23] El-Athman R, Fuhr L, Relogio A (2018). A Systems-Level Analysis Reveals Circadian Regulation of Splicing in Colorectal Cancer. EBioMedicine.

[24] Torres M, Becquet D, Franc JL, Francois-Bellan AM (2018). Circadian processes in the RNA life cycle. Wiley Interdiscip Rev RNA.

[25] El-Athman R (2017). The Ink4a/Arf locus operates as a regulator of the circadian clock modulating RAS activity. PLoS Biol.

[26] Fuhr L (2018). The Circadian Clock Regulates Metabolic Phenotype Rewiring Via HKDC1 and Modulates Tumor Progression and Drug Response in Colorectal Cancer. EBioMedicine.

[27] Abreu M, Basti A, Genov N, Mazzoccoli G, Relógio A (2018). The reciprocal interplay between TNFα and the circadian clock impacts on cell proliferation and migration in Hodgkin lymphoma cells. Scientific Reports.

[28] Relogio A (2014). Ras-mediated deregulation of the circadian clock in cancer. PLoS Genet.

[29] Lamia KA (2017). Ticking time bombs: connections between circadian clocks and cancer. F1000Res.

[30] Kim TD (2007). Rhythmic control of AANAT translation by hnRNP Q in circadian melatonin production. Genes Dev.

[31] Abreu, M., Basti, A., Genov, N. & Mazzoccoli, G. The reciprocal interplay between TNFalpha and the circadian clock impacts on cell proliferation and migration in Hodgkin lymphoma cells. **8**, 11474, 10.1038/s41598-018-29847-z (2018). 10.1038/s41598-018-29847-z PMC6068144 30065253

[32] Piva F, Giulietti M, Burini AB, Principato G (2012). SpliceAid 2: a database of human splicing factors expression data and RNA target motifs. Hum Mutat.

[33] Johannessen B, Sveen A, Skotheim RI (2015). TIN: An R Package for Transcriptome Instability Analysis. Cancer Inform.

[34] Ornitz DM, Itoh N (2015). The Fibroblast Growth Factor signaling pathway. Wiley Interdiscip Rev Dev Biol.

[35] Lehmann R (2015). Assembly of a comprehensive regulatory network for the mammalian circadian clock: a bioinformatics approach. PLoS One.

[36] Razick S, Magklaras G, Donaldson IM (2008). iRefIndex: a consolidated protein interaction database with provenance. BMC Bioinformatics.

[37] Sayed M, Pelech S, Wong C, Marotta A, Salh B (2001). Protein kinase CK2 is involved in G2 arrest and apoptosis following spindle damage in epithelial cells. Oncogene.

[38] Umemura Y (2013). An *in vitro* ES cell-based clock recapitulation assay model identifies CK2alpha as an endogenous clock regulator. PLoS One.

[39] Treiber T (2017). A Compendium of RNA-Binding Proteins that Regulate MicroRNA Biogenesis. Mol Cell.

[40] Morchikh M (2017). HEXIM1 and NEAT1 Long Non-coding RNA Form a Multi-subunit Complex that Regulates DNA-Mediated Innate Immune Response. Mol Cell.

[41] Han Z (2006). Structural basis for the specific recognition of methylated histone H3 lysine 4 by the WD-40 protein WDR5. Mol Cell.

[42] Mathew R (2008). Phosphorylation of human PRP28 by SRPK2 is required for integration of the U4/U6-U5 tri-snRNP into the spliceosome. Nat Struct Mol Biol.

[43] Ballut L (2005). The exon junction core complex is locked onto RNA by inhibition of eIF4AIII ATPase activity. Nat Struct Mol Biol.

[44] Zhang X (2017). An Atomic Structure of the Human Spliceosome. Cell.

[45] Mayeda A (1999). Purification and characterization of human RNPS1: a general activator of pre-mRNA splicing. EMBO J.

[46] Lykke-Andersen J, Shu MD, Steitz JA (2001). Communication of the position of exon-exon junctions to the mRNA surveillance machinery by the protein RNPS1. Science.

[47] Kulakovskiy IV (2018). HOCOMOCO: towards a complete collection of transcription factor binding models for human and mouse via large-scale ChIP-Seq analysis. Nucleic Acids Res.

[48] Puga A, Xia Y, Elferink C (2002). Role of the aryl hydrocarbon receptor in cell cycle regulation. Chem Biol Interact.

[49] Schulte KW, Green E, Wilz A, Platten M, Daumke O (2017). Structural Basis for Aryl Hydrocarbon Receptor-Mediated Gene Activation. Structure.

[50] Bhoumik A (2005). ATM-dependent phosphorylation of ATF2 is required for the DNA damage response. Mol Cell.

[51] Sgarbanti M, Marsili G, Remoli AL, Orsatti R, Battistini A (2007). IRF-7: new role in the regulation of genes involved in adaptive immunity. Ann N Y Acad Sci.

[52] Milanini-Mongiat J, Pouyssegur J, Pages G (2002). Identification of two Sp1 phosphorylation sites for p42/p44 mitogen-activated protein kinases: their implication in vascular endothelial growth factor gene transcription. J Biol Chem.

[53] Bonello MR, Khachigian LM (2004). Fibroblast growth factor-2 represses platelet-derived growth factor receptor-alpha (PDGFR-alpha) transcription via ERK1/2-dependent Sp1 phosphorylation and an atypical cis-acting element in the proximal PDGFR-alpha promoter. J Biol Chem.

[54] Hsu MC, Chang HC, Hung WC (2006). HER-2/neu represses the metastasis suppressor RECK via ERK and Sp transcription factors to promote cell invasion. J Biol Chem.

[55] Olofsson BA, Kelly CM, Kim J, Hornsby SM, Azizkhan-Clifford J (2007). Phosphorylation of Sp1 in response to DNA damage by ataxia telangiectasia-mutated kinase. Mol Cancer Res.

[56] Xiao J (2013). Transcription factor NF-Y is a functional regulator of the transcription of core clock gene Bmal1. J Biol Chem.

[57] Tao W (2015). EGR1 regulates hepatic clock gene amplitude by activating Per1 transcription. Sci Rep.

[58] Hughes ME (2009). Harmonics of circadian gene transcription in mammals. PLoS Genet.

[59] Wu G, Anafi RC, Hughes ME, Kornacker K, Hogenesch JB (2016). MetaCycle: an integrated R package to evaluate periodicity in large scale data. Bioinformatics.

[60] Chen T, Damaj BB, Herrera C, Lasko P, Richard S (1997). Self-association of the single-KH-domain family members Sam68, GRP33, GLD-1, and Qk1: role of the KH domain. Mol Cell Biol.

[61] Das R, Zhou Z, Reed R (2000). Functional association of U2 snRNP with the ATP-independent spliceosomal complex E. Mol Cell.

[62] Makarova OV, Makarov EM, Liu S, Vornlocher HP, Luhrmann R (2002). Protein 61K, encoded by a gene (PRPF31) linked to autosomal dominant retinitis pigmentosa, is required for U4/U6*U5 tri-snRNP formation and pre-mRNA splicing. EMBO J.

[63] David-Morrison G (2016). WAC Regulates mTOR Activity by Acting as an Adaptor for the TTT and Pontin/Reptin Complexes. Dev Cell.

[64] Kowalska E (2013). NONO couples the circadian clock to the cell cycle. Proc Natl Acad Sci USA.

[65] Kershnar E, Wu SY, Chiang CM (1998). Immunoaffinity purification and functional characterization of human transcription factor IIH and RNA polymerase II from clonal cell lines that conditionally express epitope-tagged subunits of the multiprotein complexes. J Biol Chem.

[66] Clark VE (2016). Recurrent somatic mutations in POLR2A define a distinct subset of meningiomas. Nat Genet.

[67] Errico A (2015). Colorectal cancer: POLR2A deletion with TP53 opens a window of opportunity for therapy. Nat Rev Clin Oncol.

[68] Romero JP (2016). EventPointer: an effective identification of alternative splicing events using junction arrays. BMC Genomics.

[69] McDowell IC (2018). Clustering gene expression time series data using an infinite Gaussian process mixture model. PLoS Comput Biol.

[70] Golumbeanu M (2019). Proteo-Transcriptomic Dynamics of Cellular Response to HIV-1 Infection. Sci Rep.

[71] TMixClust: Time Series Clustering of Gene Expression with Gaussian Mixed-Effects Models and Smoothing Splines v. 1.6.0 (Bioconductor, 2019).

[72] Thaben PF, Westermark PO (2014). Detecting rhythms in time series with RAIN. J Biol Rhythms.

[73] Takenaka S (2010). Downregulation of SS18-SSX1 expression in synovial sarcoma by small interfering RNA enhances the focal adhesion pathway and inhibits anchorage-independent growth *in vitro* and tumor growth *in vivo*. Int J Oncol.

[74] Sastry KS (2017). Cytoprotective effect of neuropeptides on cancer stem cells: vasoactive intestinal peptide-induced antiapoptotic signaling. Cell Death Dis.

[75] Broderick P (2010). IRF4 polymorphism rs872071 and risk of Hodgkin lymphoma. Br J Haematol.

[76] Aldinucci D, Celegato M, Borghese C, Colombatti A, Carbone A (2011). IRF4 silencing inhibits Hodgkin lymphoma cell proliferation, survival and CCL5 secretion. Br J Haematol.

[77] Naaby-Hansen S (2002). CABYR, a novel calcium-binding tyrosine phosphorylation-regulated fibrous sheath protein involved in capacitation. Dev Biol.

[78] Hsu HC (2005). Characterization of two non-testis-specific CABYR variants that bind to GSK3beta with a proline-rich extensin-like domain. Biochem Biophys Res Commun.

[79] Clark J (1994). Identification of novel genes, SYT and SSX, involved in the t(X;18)(p11.2;q11.2) translocation found in human synovial sarcoma. Nat Genet.

[80] Lee, A. M. *et al*. DPYD variants as predictors of 5-fluorouracil toxicity in adjuvant colon cancer treatment (NCCTG N0147). J Natl Cancer Inst **106**, 10.1093/jnci/dju298 (2014). 10.1093/jnci/dju298 PMC4271081 25381393

[81] Gotoh T (2016). Model-driven experimental approach reveals the complex regulatory distribution of p53 by the circadian factor Period 2. Proc Natl Acad Sci USA.

[82] Sporl F (2011). A circadian clock in HaCaT keratinocytes. J Invest Dermatol.

[83] Livak KJ, Schmittgen TD (2001). Analysis of relative gene expression data using real-time quantitative PCR and the 2(-Delta Delta C(T)) Method. Methods.

[84] Yu G, Wang LG, Han Y, He Q (2012). Y. clusterProfiler: an R package for comparing biological themes among gene clusters. OMICS.

[85] Yu G, He QY (2016). ReactomePA: an R/Bioconductor package for reactome pathway analysis and visualization. Mol Biosyst.

[86] Bailey TL, Johnson J, Grant CE, Noble WS (2015). The MEME Suite. Nucleic Acids Res.

[87] Shannon P (2003). Cytoscape: a software environment for integrated models of biomolecular interaction networks. Genome Res.

[88] Mora A, Donaldson IM (2011). iRefR: an R package to manipulate the iRefIndex consolidated protein interaction database. BMC Bioinformatics.

[89] Morris JH (2011). clusterMaker: a multi-algorithm clustering plugin for Cytoscape. BMC Bioinformatics.

[90] Gentleman RC (2004). Bioconductor: open software development for computational biology and bioinformatics. Genome Biol.

[91] Pizarro A, Hayer K, Lahens NF, Hogenesch JB (2013). CircaDB: a database of mammalian circadian gene expression profiles. Nucleic Acids Res.

[92] Durinck S, Bullard J, Spellman PT, Dudoit S (2009). GenomeGraphs: integrated genomic data visualization with R. BMC Bioinformatics.

[93] Luck S, Thurley K, Thaben PF, Westermark PO (2014). Rhythmic degradation explains and unifies circadian transcriptome and proteome data. Cell Rep.

