# Supplementary material for: A bioinformatic analysis identifies circadian expression of splicing factors and time-dependent alternative splicing events in the HD-MY-Z cell line
Source: Sci Rep. 2019 Jul 30;9:11062. doi: 10.1038/s41598-019-47343-w (PMC6667479; doi:10.1038/s41598-019-47343-w)
Supplement: Supplementary file 1 — Supplementary Information [file 41598_2019_47343_MOESM1_ESM.pdf]

Supplementary Information

A bioinformatics analysis identifies circadian expression of splicing factors and time-dependent alternative splicing events in the HD-MY-Z cell line

Nikolai Genov[1,2], Alireza Basti[1,2], Mónica Abreu[1,2], Rosario Astaburuaga[1,2], Angela Relógio[1,2,*]

[1]Charité - Universitätsmedizin Berlin, corporate member of Freie Universität Berlin, Humboldt - Universität zu Berlin, and Berlin Institute of Health, Institute for Theoretical Biology, Germany

[2]Charité - Universitätsmedizin Berlin, corporate member of Freie Universität Berlin, Humboldt - Universität zu Berlin, and Berlin Institute of Health, Medical Department of Hematology, Oncology, and Tumor Immunology, Molecular Cancer Research Center, Germany

[*]Corresponding author: angela.relogio@charite.de

Supplementary Materials

Supplementary Figure S1 - High-resolution representation of the network of SF clustered in 9 groups based on their protein-protein interaction data. Depicted is the protein-protein interaction (PPI) network generated from the list of SF. The network was created based on PPI data retrieved from the IntAct database and subsequently reduced to the main connected components of each element. The clusterMaker Cytoscape plugin was used for generating the clustering with a community clustering algorithm (GLay) resulting in the nine clusters shown. The clusters contain a total of 237 nodes and 898 edges.

Supplementary Figure S2 - High-resolution representation of the network of SF and NCRG points towards a possible interaction between the two sets. The network was generated based on the PPI data retrieved form the IntAct database. The network consists of 251 SF (red) and 130 NCRG elements (blue) in a total of 186 protein-protein interactions between both groups.

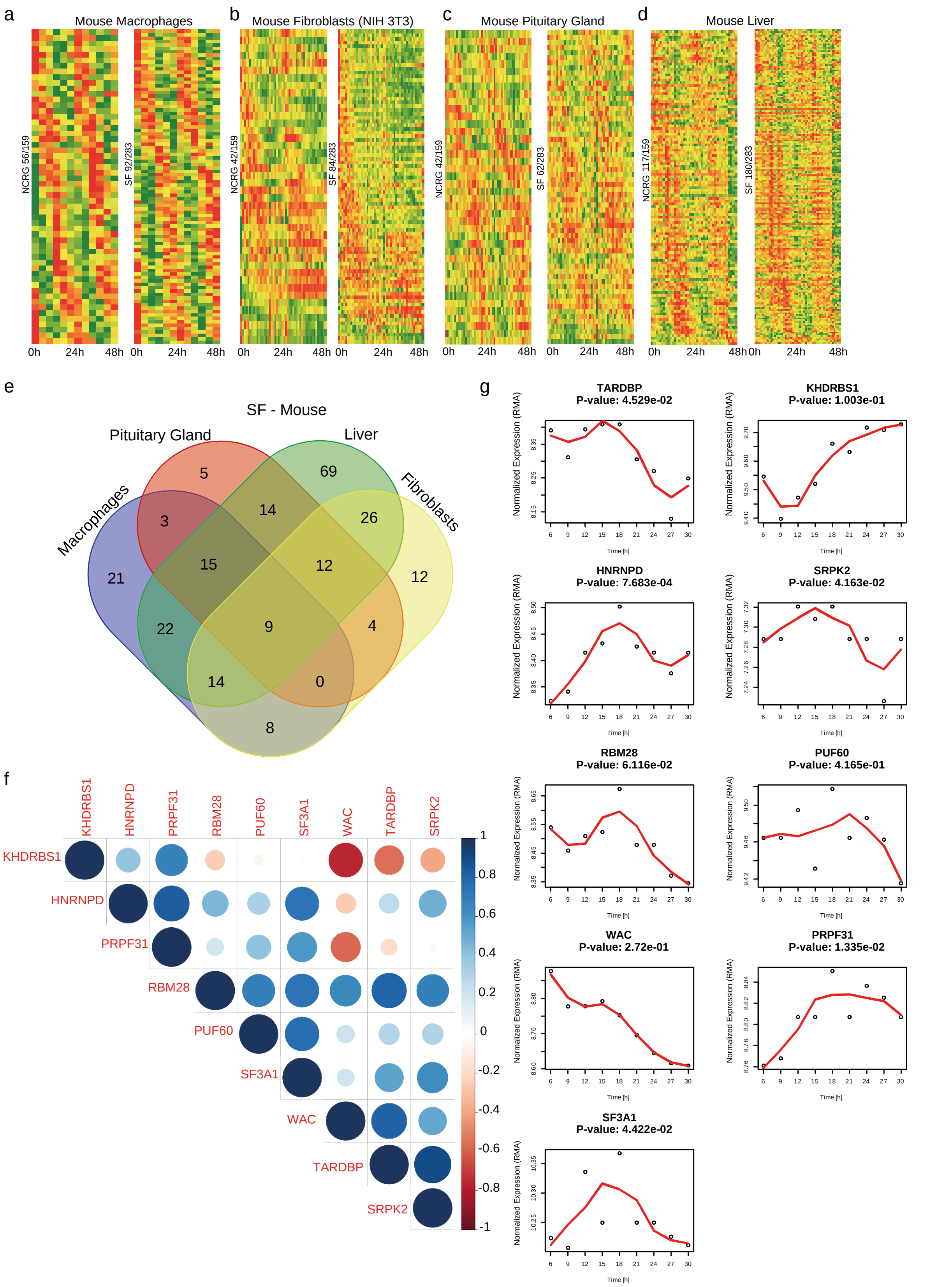

Supplementary Figure S3 – Analysis of circadian rhythmic genes in different murine tissues for the NCRG and the SF predefined sets. (a-d) Elements of the NCRG and the SF sets oscillate in mouse macrophages, fibroblasts, pituitary gland and liver. The number of

oscillating genes varies across tissues. The total number of genes for the NCRG and the SF is derived after mapping the human Entrez Gene IDs to the mouse Entrez Gene IDs. **(e)** A comparison among oscillating SF for the mouse cell lines shows an intersection of 9 genes: *Hnrnpd*, *Khdrbs1*, *Srpk2*, *Wac*, *Tardbp*, *Sf3a1*, *Puf60*, *Rbm28*, *Prpf31*. **(f)** The gene expression of the nine SF detected to oscillate in mouse samples with a period between 21 and 27h shows a high positive (blue)/negative (red) correlation in the microarray data set for the human HD-MY-Z cell line. **(g)** Depicted are the normalized expression values for the nine SF, retrieved from the HD-MY-Z microarray data set. Among these nine SF, several genes oscillate (p < 0.05) in the HD-MY-Z as well and TARDP oscillates also in the U2OS human cell line (**Figure S4**). All p-values were calculated using MetaCycle.

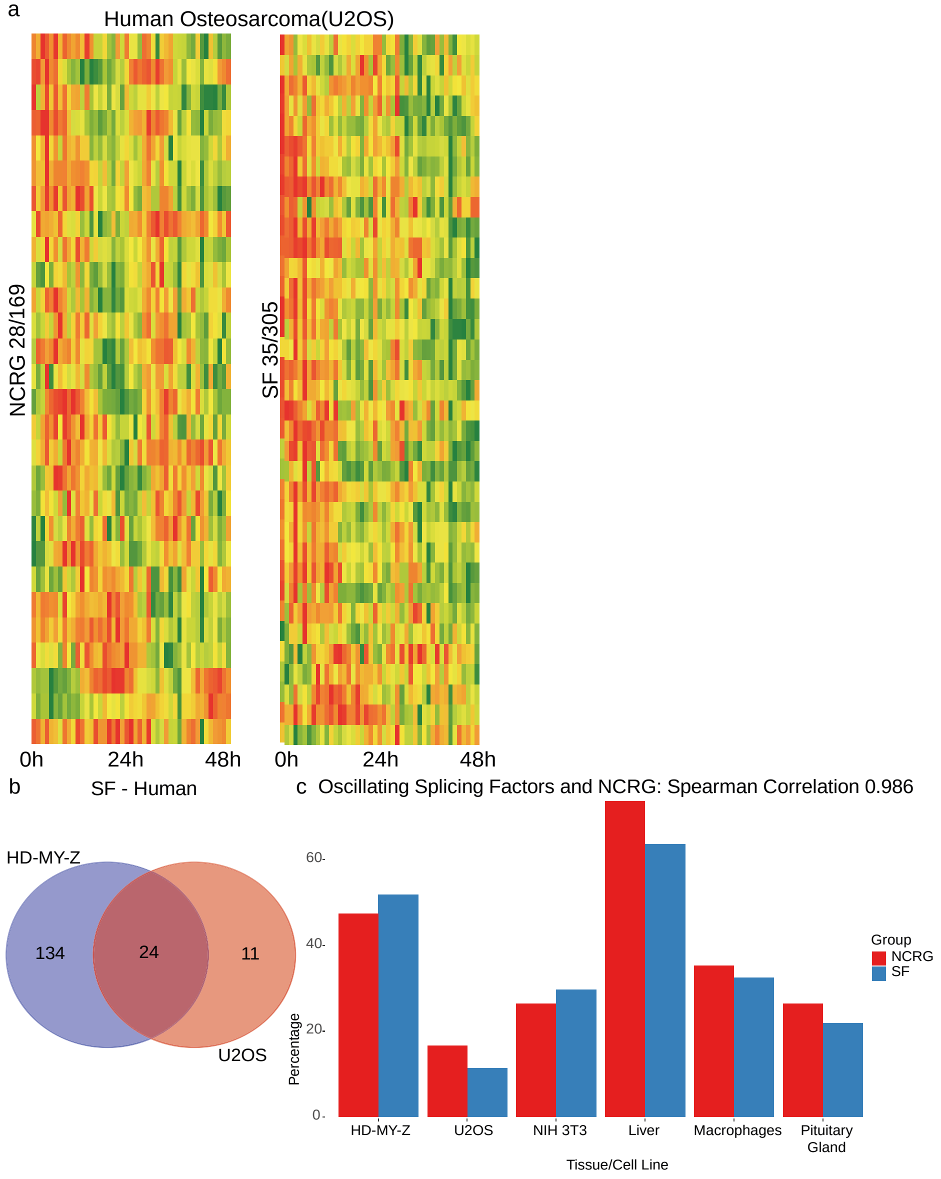

Supplementary Figure S4 – A comparison of oscillating genes in the NCRG and the SF sets across mammalian tissues and two cancer cell lines points to a correlation between both sets. (a) We analysed a time course data set (see Materials and Methods) for the human osteosarcoma cell line (U2OS) using MetaCycle to determine the oscillating SF and NCRG elements. 28 NCRG and 35 SF elements show oscillations with a period between 21 and 27 hours. **(b)** There is an overlap of 24 SF oscillating with a period of 21 to 27 hours in the HD-MY-Z cell line and the U2OS cell line. **(c)** We compared the number of oscillating SF and oscillating NCRG genes between all samples which resulted in a spearman correlation of 0.986.

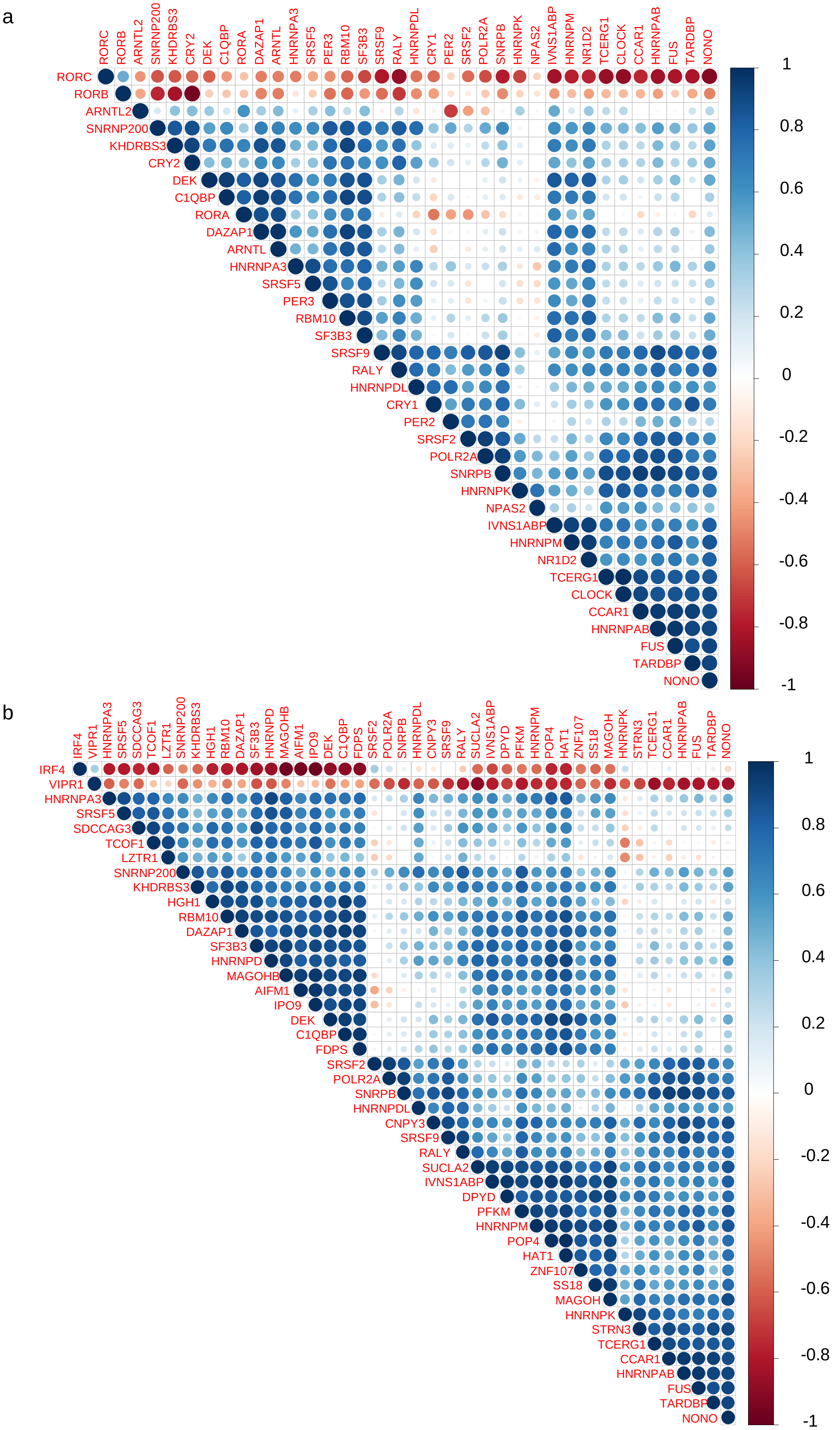
a
RORC RORB ARNTL2 SNRNP200 KHDRBS3 CRY2 DEK C1QBP RORA DAZAP1 ARNTL HNRNPA3 SRSF5 PER3 RBM10 SF3B3 SRSF9 RALY HNRNPDL CRY1 PER2 SRSF2 POLR2A SNRPB HNRNPK NPAS2 IVNS1ABP HNRNPM NR1D2 TCERG1 CLOCK CCAR1 HNRNPAB FUS TARDBP NONO
1 0.8 0.6 0.4 0.2 0 -0.2 -0.4 -0.6 -0.8 -1
b
IRF4 VIPR1 HNRNPA3 SRSF5 SDCCAG3 TCOF1 LZTR1 SNRNP200 KHDRBS3 HGH1 RBM10 DAZAP1 SF3B3 HNRNPD MAGOHB AIFM1 IPO9 DEK C1QBP FDPS SRSF2 POLR2A SNRPB HNRNPDL CNPY3 SRSF9 RALY SUCLA2 IVNS1ABP DPYD PFKM HNRNPM POP4 HAT1 ZNF107 SS18 MAGOH HNRNPK STRN3 TCERG1 CCAR1 HNRNPAB FUS TARDBP NONO
1 0.8 0.6 0.4 0.2 0 -0.2 -0.4 -0.6 -0.8 -1

Supplementary Figure S5 – The 24 SF oscillating in U2OS and HD-MY-Z show high correlations with the core-clock network and the alternatively spliced genes. (a) Depicted is a comparison between the core-clock network composed of 12 elements and the set of 24 SF for which oscillations with a period between 21h and 27h were detected in the U2OS and HD-MY-Z cell lines. The correlation of expression between the two sets shows consistent negative correlations (red) between RORC/RORB and most of the other genes and positive correlations (blue) between the other elements of the core-clock network and the 24 oscillating SF. **(b)** Depicted is a comparison between the 24 SF and the 21 genes for which splicing events were detected and which are also detected to oscillate with a period between 21h and 27h. The plots show an overall positive correlation (blue) between the expression of the genes in the two groups.

a

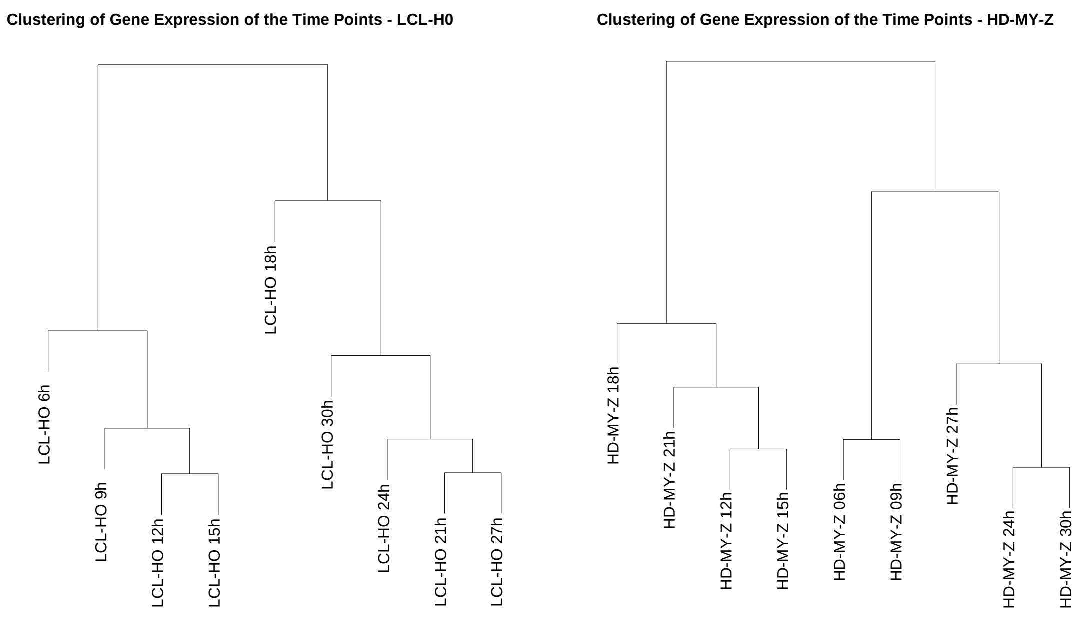

b

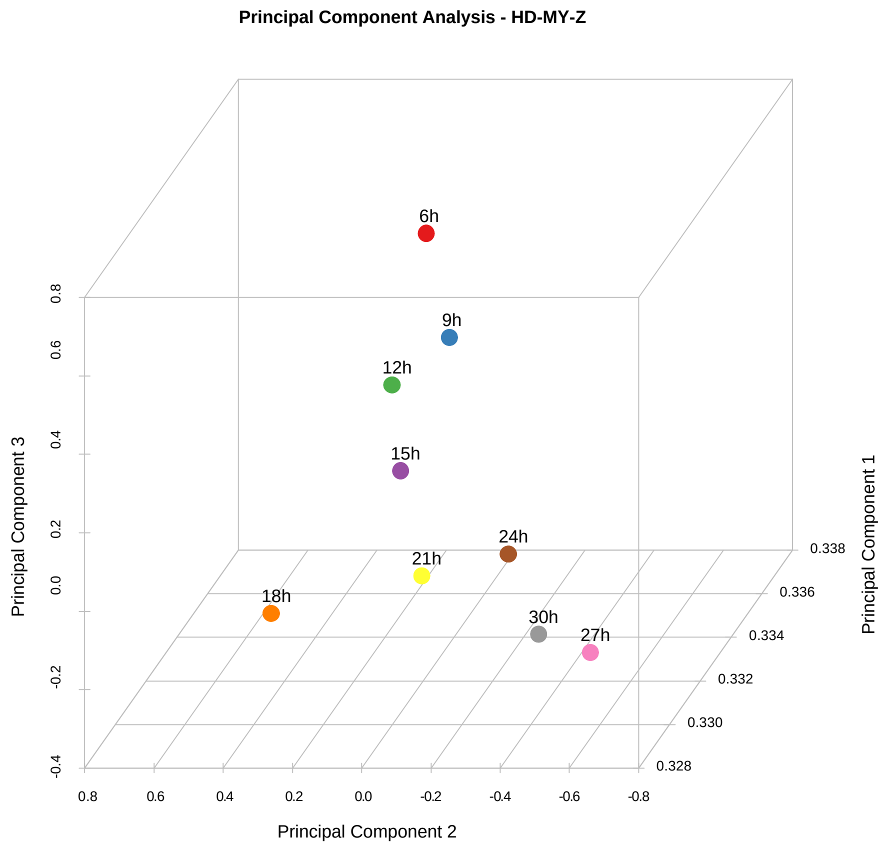

Supplementary Figure S6 - Clustering and PCA of gene-level expression for the LCL-HO cell line and the HD-MY-Z cell line data set identify the 18h time point as having the largest distance to the other time points. Both the hierarchical clustering computed with the hclust

function and the “complete” method in R[1] **(a)** and principle component analysis computed with princomp also in R **(b)** show the 18h point as having the largest distance to the other points. Given the pairwise analysis design of the test for alternative splicing performed with EventPointer the 18h time point was excluded from subsequent analysis for identification of genes with alternative splicing events. Should the 18h time point be paired with any other point, the difference between the points in the pair would be larger than the differences between the pairs. The 18h time point did however pass the tests by ArrayQualitymetrics and the expression was thus left in the visualization. The grouping of time points is known as binning or clustering of time series data in this context. Numerous publications deal with the various methods used and the advantages in terms of, for example, signal to noise ratio. Additionally, the RAIN5 R Bioconductor package commonly used in the field of circadian biology also utilizes binning to merge time points. In the original publication the authors of RAIN specifically only compare binned time points only corresponding to a raising or falling pattern[2-5].

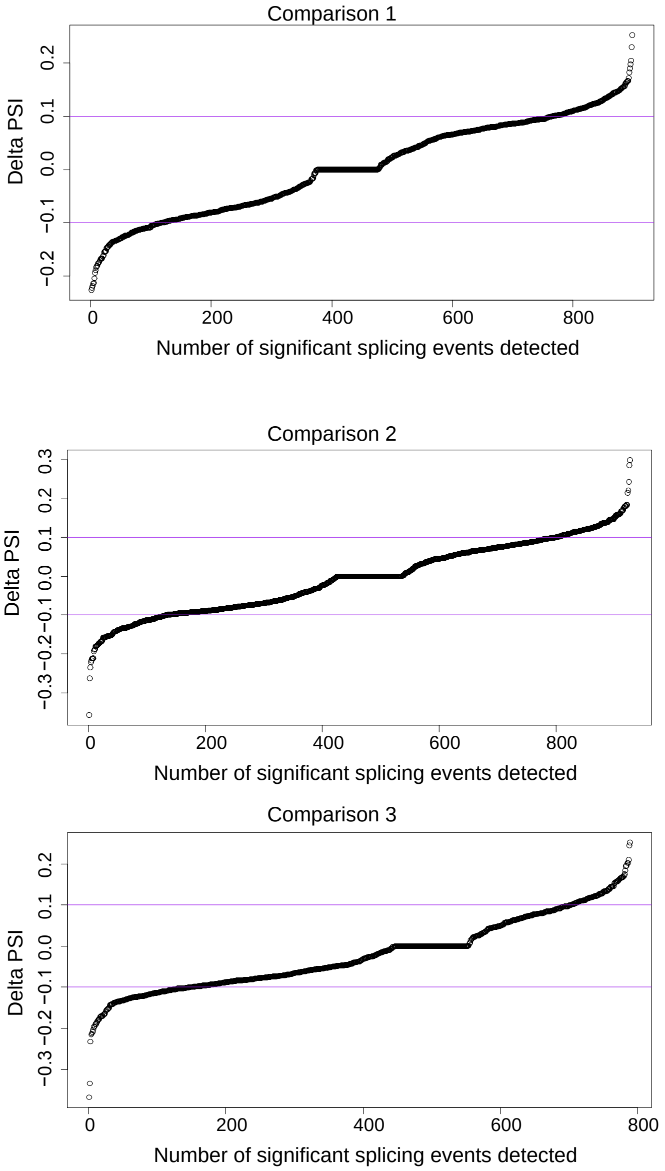

Supplementary Figure S7 - Visualization of the distribution of the delta-PSI values for the three comparisons described in Figure 4 (main text) points to a low number of events with high delta-PSI. Genes with events with |delta-PSI| > 0.1 and $p < 0.01$ were further investigated. We computed the delta-PSI with the EventPointer R package and according to the experimental design depicted in **Figure 4**. The plots show that only a few splicing events produce a high absolute value for the delta-PSI. A high delta PSI value means an increasing imbalance between the expression of two isoforms of one gene in one comparison between two pairs of timepoints. A gene can be represented by more than one data point due to the large number of isoforms.

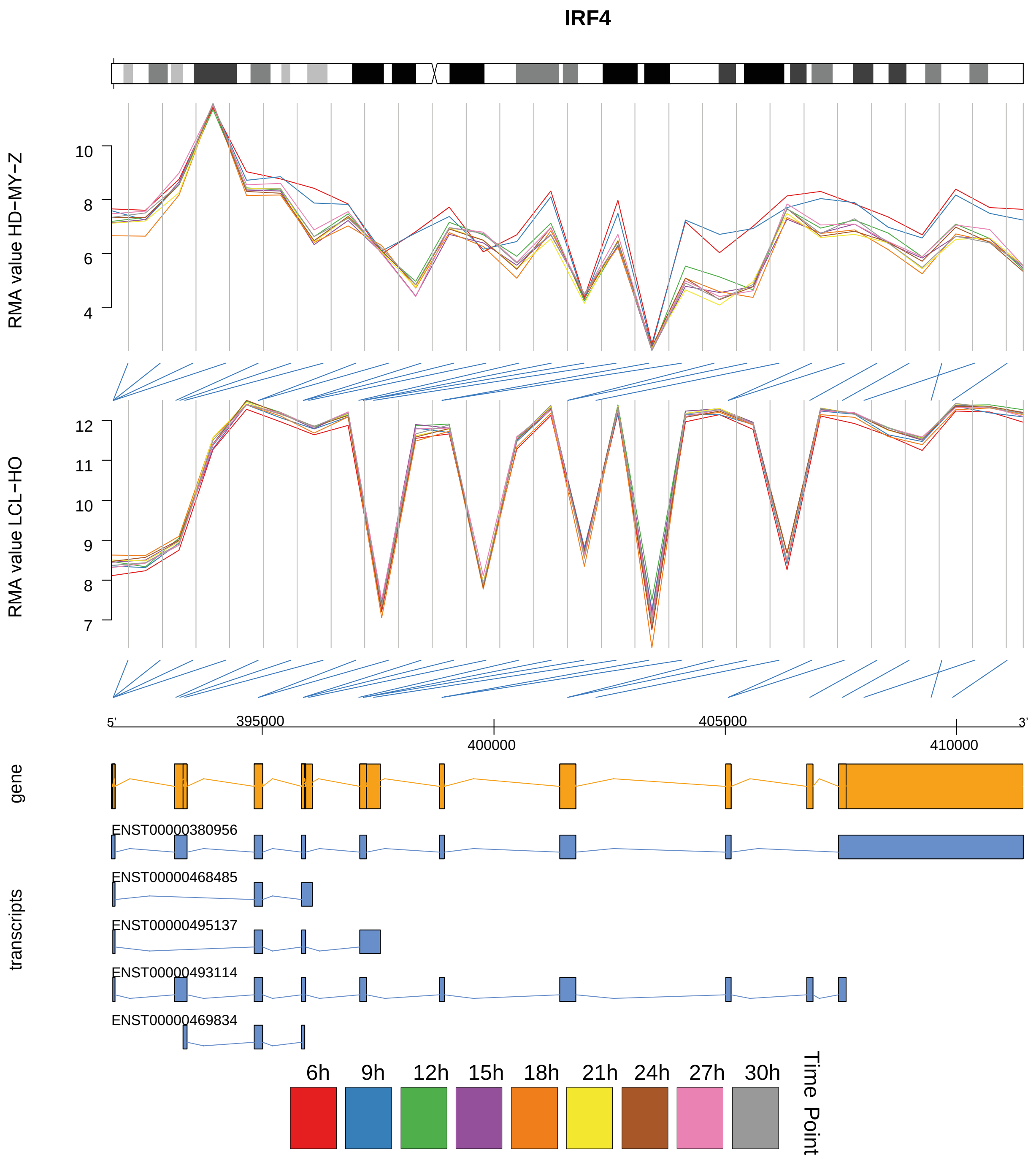

Supplementary Figure S8 - Detailed comparison of the probeset level expression of the gene IRF4 between the cell lines HD-MY-Z and LCL-HO shows localized fluctuations of expression pointing to the existence of splicing events. Depicted are the expression of the corresponding probesets for IRF4 for the HD-MY-Z and the LCL-HO cell line as a comparison. A large difference in the expression levels is present between the 6h/9h (red/blue) time points and the rest of the time points. The difference in expression between the time points

is most pronounced towards the second half of the genomic structure, notably also affecting an exon segment present in only one known isoform of the IRF4 gene – ENST00000380956. This points to an alternative splicing event at an exactly located area of the gene and at a specific temporal interval. This event can be associated with the two longest isoforms of the gene since only those contain expressed elements for the corresponding genomic region. Splicing events for the gene IRF4 in the HD-MY-Z cell line were detected using EventPointer.

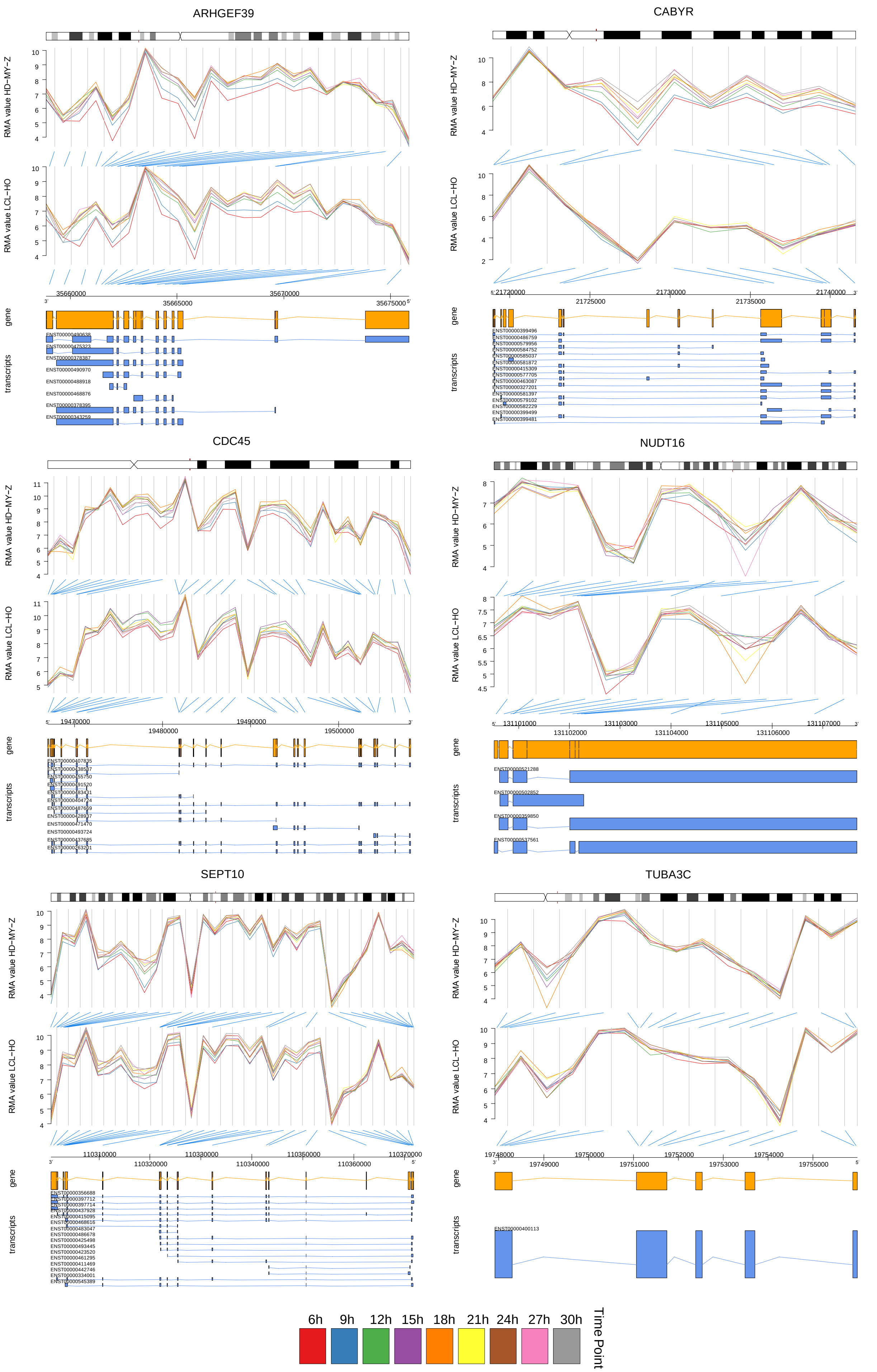
ARHGEF39
CABYR
CDC45
NUDT16
SEPT10
TUBA3C
RMA value HD−MY−Z
RMA value LCL−HO
gene
transcripts
6h
9h
12h
15h
18h
21h
24h
27h
30h
Time Point

Supplementary Figure S9 - Detailed comparison of the probeset level expression of the genes CABYR, ARHGEF39, NUDT16, SEPT10, CDC45 and TUBA3C between the cell lines HD-MY-Z and LCL-HO. We made an additional comparison (using EventPointer) between the expression levels in the HD-MY-Z and LCL-HO cell line for selected genes for which alternative splicing events were predicted in at least one of the cell lines. Both large differences (CABYR) as well as similarities (ARHGEF39) between the splicing events can be seen.

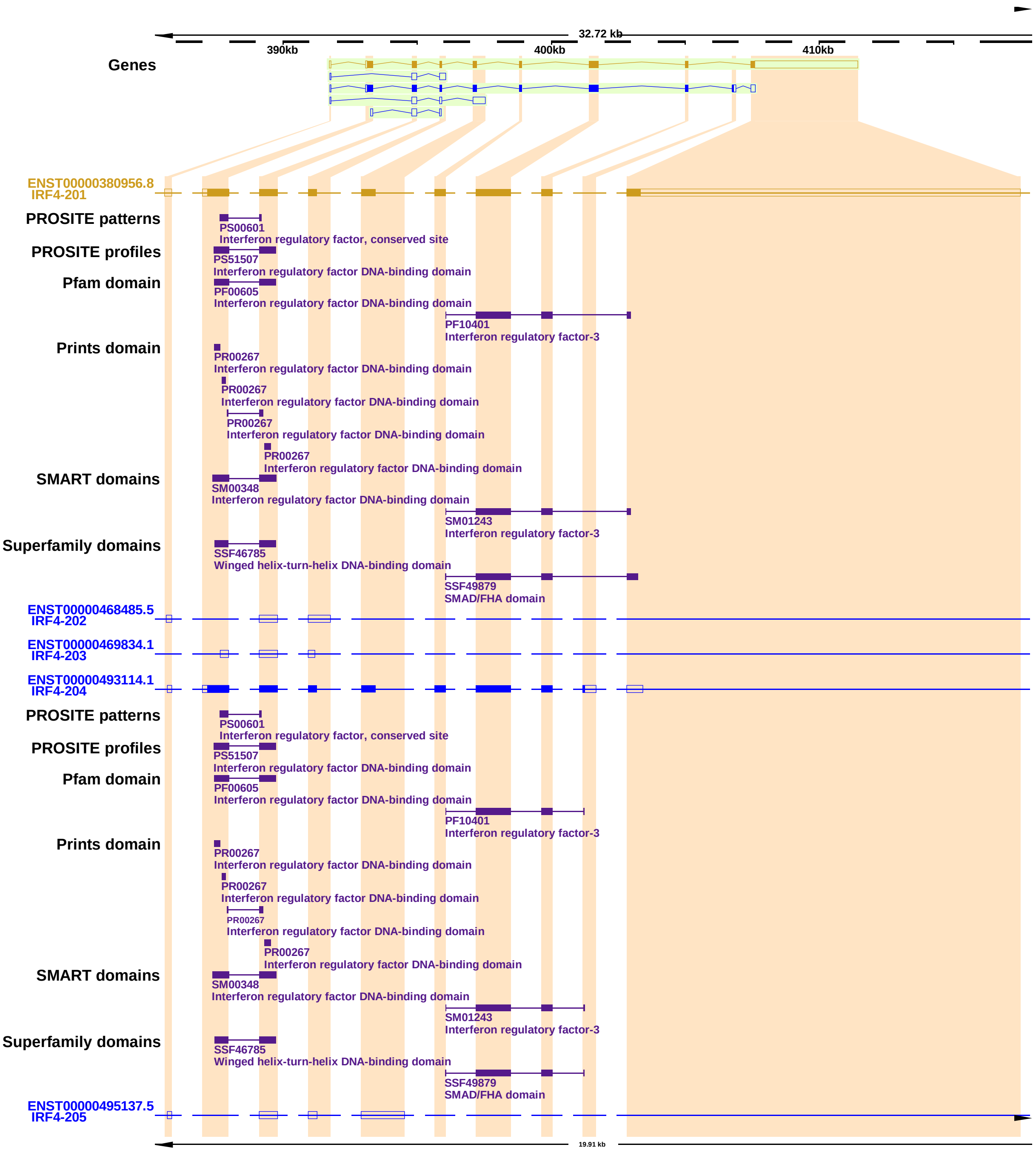

Supplementary Figure S10 – Structure of the *IRF4* gene showing its five known transcripts and the two protein coding isoforms, as well as all the associated domains. The ENST00000380956 isoform differs from the ENST00000493114 isoform in several important features. The domain for the interferon regulatory factor 3 and the SMAD/FHA domain in the shorter isoform (ENST00000493114) are incomplete. Furthermore, the short isoform undergoes nonsense-mediated decay (NMD).

Supplementary Figure S11 - Detailed network of the SF, NCRG and alternatively spliced genes. We constructed an additional network of SF, NCRG and genes for which alternative splicing events were detected. The network was created in Cytoscape from data present in the EBI IntAct database and includes the previous network of SF and NCRG (Supplemental Fig. S2). The network allows to represent visually the oscillations of expression of the genes between the timepoints 6h, 18h and 30h. We added this visualization to **Fig. 6c-e** (main text).

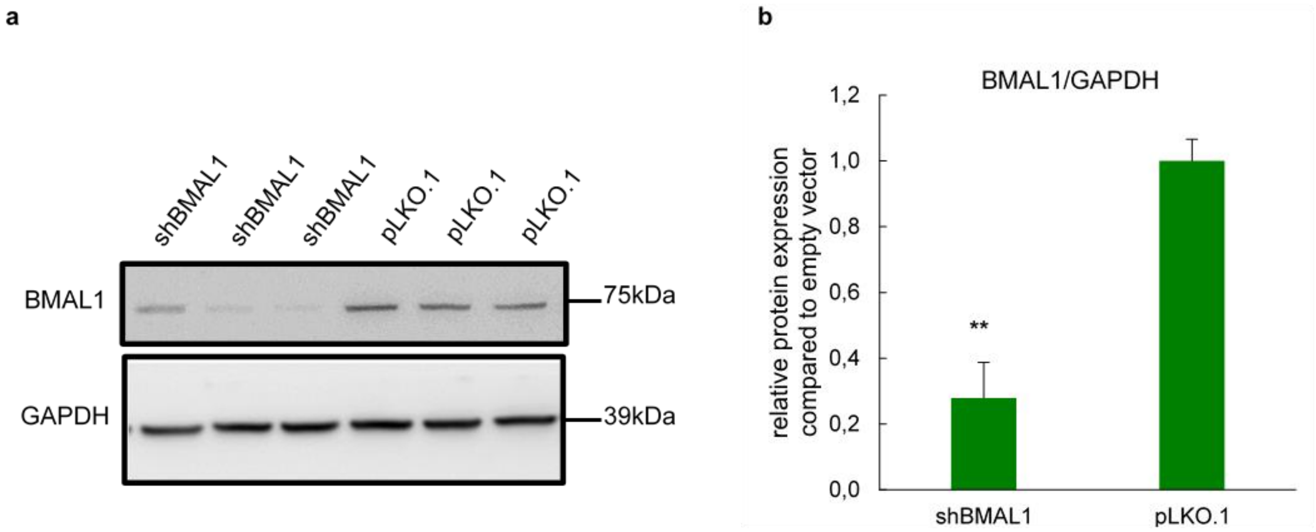

Supplementary Figure S12 – Protein levels of BMAL1 in shBMAL1 samples *vs* control. (a) Representative images of western blotting for BMAL1 protein in HD-MY-Z cells with empty vector (pLKO.1) and BMAL1 knockdown (shBMAL1). b) Quantitation of the blot band density of the BMAL1 protein in HD-MY-Z knockdown cells (shBMAL1) compared to the empty vector (pLKO.1), (mean, SEM, n = 3). **p < 0.01 by t-test. Image quantification was performed using ImageJ.

SUPPLEMENTARY TABLES

Table S1 - List of SF used as an input for the analysis.

Table S2 - Detailed table of the SF network (as depicted in **Fig. S1**). Created from IntAct data, containing all nodes. The table contains the corresponding gene symbols as well as Uniprot identifiers and additional GO/reference information for the nodes.

Table S3 - Enrichment of Reactome Pathways for the clusters of the network of SF (**Fig. S1**) created with ReactomePA and clusterProfiler with a $p < 0.05$.

Table S4 - Transcription factors detected to be enriched in the promoter regions of the SF with the AME tool of the MEME suite[2] with corresponding p-values adjusted with Bonferonni correction.

Table S5 - Enrichment of Reactome Pathways associated with the TFs for which enriched binding sites were detected in the promoter regions of the SF.

Table S6 - Alternative splicing events detected after filtering with a $p < 0.01$ and delta-PSI < 0.1 for the HD-MY-Z cell line, Comparison 1.

Table S7 - Alternative splicing events detected after filtering with a $p < 0.01$ and delta-PSI < 0.1 for the HD-MY-Z cell line, Comparison 2.

Table S8 - Alternative splicing events detected after filtering with a $p < 0.01$ and delta-PSI < 0.1 for the HD-MY-Z cell line, Comparison 3.

Table S9 - List of 42 common genes with splicing events in each of the three comparisons with $p < 0.01$ that show the greatest variance of delta-PSI over time.

Table S10 - Enriched Reactome pathways for the genes undergoing alternative splicing in Comparison 1, Comparison 2 and Comparison 3.

Table S11 – Circadian parameters for a variable period (period, p-value, amplitude, acrophase, and acrophase shift between control and shBmal1 condition) for HNRNPAB, SRSF5, POP4, DPYD, and IRF4 in control and shBmal1 condition.

Gene	Condition	Period [h]	Amplitude [a.u.]	p-value	Acrophase [h]	Acrophase shift (ctrl - sh*Bmal1*) [h]
**HNRNPAB**	**ctrl**	27.0	0.20	2.82E-02	16.72	2.99
	**sh*Bmal1***	27.0	0.31	1.14E-04	13.73	
**SRSF5**	**ctrl**	18.7	0.19	7.28E-03	18.72	3.30
	**sh*Bmal1***	20.8	0.20	1.09E-03	15.42	
**POP4**	**ctrl**	27.0	0.15	2.64E-02	14.89	0.11
	**sh*Bmal1***	27.0	0.26	2.35E-05	14.77	
**DPYD**	**ctrl**	14.0	0.12	3.07E-02	15.16	0.51
	**sh*Bmal1***	25.1	0.14	3.19E-03	14.65	
**IRF4**	**ctrl**	27.0	0.33	9.63E-05	14.69	2.17
	**sh*Bmal1***	27.0	0.35	1.67E-02	12.52	

Table S12 - *Circadian parameters for a 24h-period (p-value of the model fit, amplitude, acrophase, and acrophase shift between control and shBmal1 condition) for HNRNPAB, SRSF5, POP4, DPYP, and IRF4 in control and shBmal1 condition.*

Gene	Condition	Period [h]	Amplitude [a.u.]	p-value	Acrophase [h]	Acrophase shift (ctrl - sh*Bmal1*) [h]
**HNRNPAB**	**ctrl**	24.0	0.19	3.28E-02	16.85	2.51
	**sh*Bmal1***	24.0	0.31	5.41E-04	14.33	
**SRSF5**	**ctrl**	24.0	0.19	8.29E-03	19.77	5.03
	**sh*Bmal1***	24.0	0.18	2.49E-03	14.74	
**POP4**	**ctrl**	24.0	0.14	4.86E-02	15.37	0.12
	**sh*Bmal1***	24.0	0.25	2.13E-04	15.26	
**DPYD**	**ctrl**	24.0	0.08	2.69E-01	26.15	11.32
	**sh*Bmal1***	24.0	0.14	3.32E-03	14.82	
**IRF4**	**ctrl**	24.0	0.33	2.26E-04	15.05	1.89
	**sh*Bmal1***	24.0	0.35	2.79E-02	13.15	

References

1 R: A language and environment for statistical computing. v. 3.5.3 (R Foundation for Statistical Computing, 2019).

2 Bailey, T. L., Johnson, J., Grant, C. E. & Noble, W. S. The MEME Suite. *Nucleic Acids Res* **43**, W39-49, doi:10.1093/nar/gkv416 (2015).
