# Supplementary material for: A bioinformatic analysis identifies circadian expression of splicing factors and time-dependent alternative splicing events in the HD-MY-Z cell line
Source: Sci Rep. 2019 Jul 30;9:11062. doi: 10.1038/s41598-019-47343-w (PMC6667479; doi:10.1038/s41598-019-47343-w)

Supplementary Information

A bioinformatics analysis identifies circadian expression of splicing factors and time-dependent alternative splicing events in the HD-MY-Z cell line

Nikolai Genov[1,2], Alireza Basti[1,2], Mónica Abreu[1,2], Rosario Astaburuaga[1,2], Angela Relógio[1,2,*]

[1]Charité - Universitätsmedizin Berlin, corporate member of Freie Universität Berlin, Humboldt - Universität zu Berlin, and Berlin Institute of Health, Institute for Theoretical Biology, Germany

[2]Charité - Universitätsmedizin Berlin, corporate member of Freie Universität Berlin, Humboldt - Universität zu Berlin, and Berlin Institute of Health, Medical Department of Hematology, Oncology, and Tumor Immunology, Molecular Cancer Research Center, Germany

[*]Corresponding author: angela.relogio@charite.de

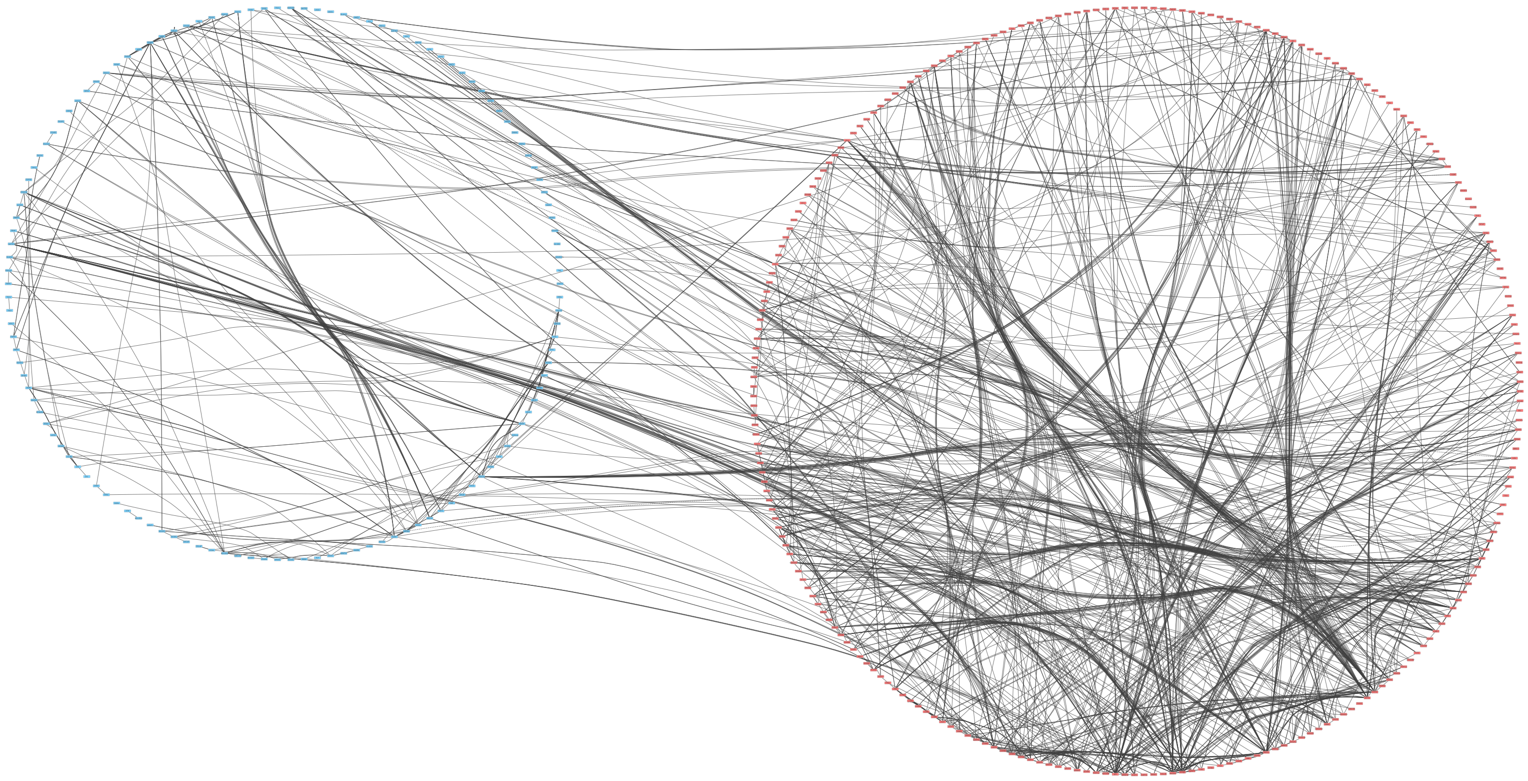

Supplement: Supplementary file 3 — Figure S2 [file 41598_2019_47343_MOESM3_ESM.pdf]
